# Research on impact dynamic characteristics of hydraulic column cylinder barrel

**DOI:** 10.1371/journal.pone.0348487

**Published:** 2026-11-06

**Authors:** Jianzhuo Zhang, Tao Wang, Wenliang Li, Chuanxu Wan, Hao Guo

**Affiliations:** 1 School of Mechanical Engineering, Liaoning Technical University, Fuxin City, Liaoning, China; 2 Shandong Yankuang Intelligent Manufacturing Co., Zoucheng City, Shandong, China; 3 Xinjiang Tianchi Energy Co., Ltd., Changji Hui Autonomous Prefecture City, Xinjiang, China; NED University of Engineering and Technology, PAKISTAN

## Abstract

During the loading process of a hydraulic column, the magnitude and distribution of stress in the cylinder barrel are critical to the safety of the hydraulic column. Aiming at the problem of stress distribution in the cylinder barrel of a hydraulic column under liquid pressure, a mathematical model of cylinder barrel stress is established based on the thick-walled cylinder theory and the fundamental theory of rotating shells. It is concluded that the main factors affecting the stress distribution of the hydraulic column cylinder barrel are the inner diameter of the cylinder barrel, the wall thickness of the cylinder barrel, and the height of the rodless chamber of the column. Numerical simulation, response surface methodology, and experimental research are adopted. The results show that the stress of the hydraulic column cylinder barrel exhibits an “M-shaped” distribution, with the stress peak located at the bottom of the cylinder barrel and near the piston. Among the selected structural parameters, the difference between the stress peak of the cylinder barrel and the stress valley at the cavity is most significantly affected by the height of the rodless chamber, followed by the inner diameter of the cylinder barrel, while the influence of the cylinder barrel wall thickness is the smallest. Through the response surface prediction model, it is found that when the inner diameter of the cylinder barrel is 230 mm, the wall thickness is 20 mm, and the height of the rodless chamber is 795 mm, the “M-shaped” characteristic of the stress distribution in the cylinder barrel becomes more obvious, which provides useful guidance for the design and safety assessment of hydraulic columns.

## 1. Introduction

With the continuous increase in coal mining depth, the deep geological environment is characterized by a significant rise in in-situ stress, which leads to an increased frequency of dynamic disasters such as rock bursts, roof falls and rib spalling [1–4]. These phenomena pose severe challenges to the safe production of coal mines [5–8]. As the core equipment for working face and roadway support, the reliability of hydraulic supports is critical to ensuring the safety of mining operations [9–13]. As the main load-bearing component of hydraulic supports, the structural stability of hydraulic columns under high in-situ stress and impact dynamic loads is a key factor determining the working performance of the supports [14]. Practice shows that the load-bearing capacity of hydraulic supports is mainly realized by hydraulic columns [15–18]. In the event of roof falls, rib spalling or rock bursts, the safety and reliability of the columns directly ensure the stable bearing of roof and floor pressure by the supports [19]. However, under long-term high-stress conditions, especially under repeated impact loads, cylinder expansion is prone to occur in the column barrel [20–24]. During the continuous deformation of the cylinder block, the weld joint of the outer weldment is susceptible to cracking failure under the combined superposition of circumferential tensile stress, axial tensile stress, and welding residual stress, as illustrated in Fig 1. This failure mode further induces leakage of high-pressure medium and a drastic reduction in structural load-bearing capacity, which not only constitutes a typical failure mechanism limiting the structural safety and reliability of the cylinder but also poses a significant potential hazard to the safe production of coal mines. Therefore, conducting mechanical characteristic analysis of the cylinder barrel and investigating the occurrence mechanism of cylinder expansion under high ground stress and impact dynamic load conditions holds substantial theoretical and practical significance for the development of impact-resistant hydraulic columns adaptable to complex deep mining environments.

**Fig 1:**
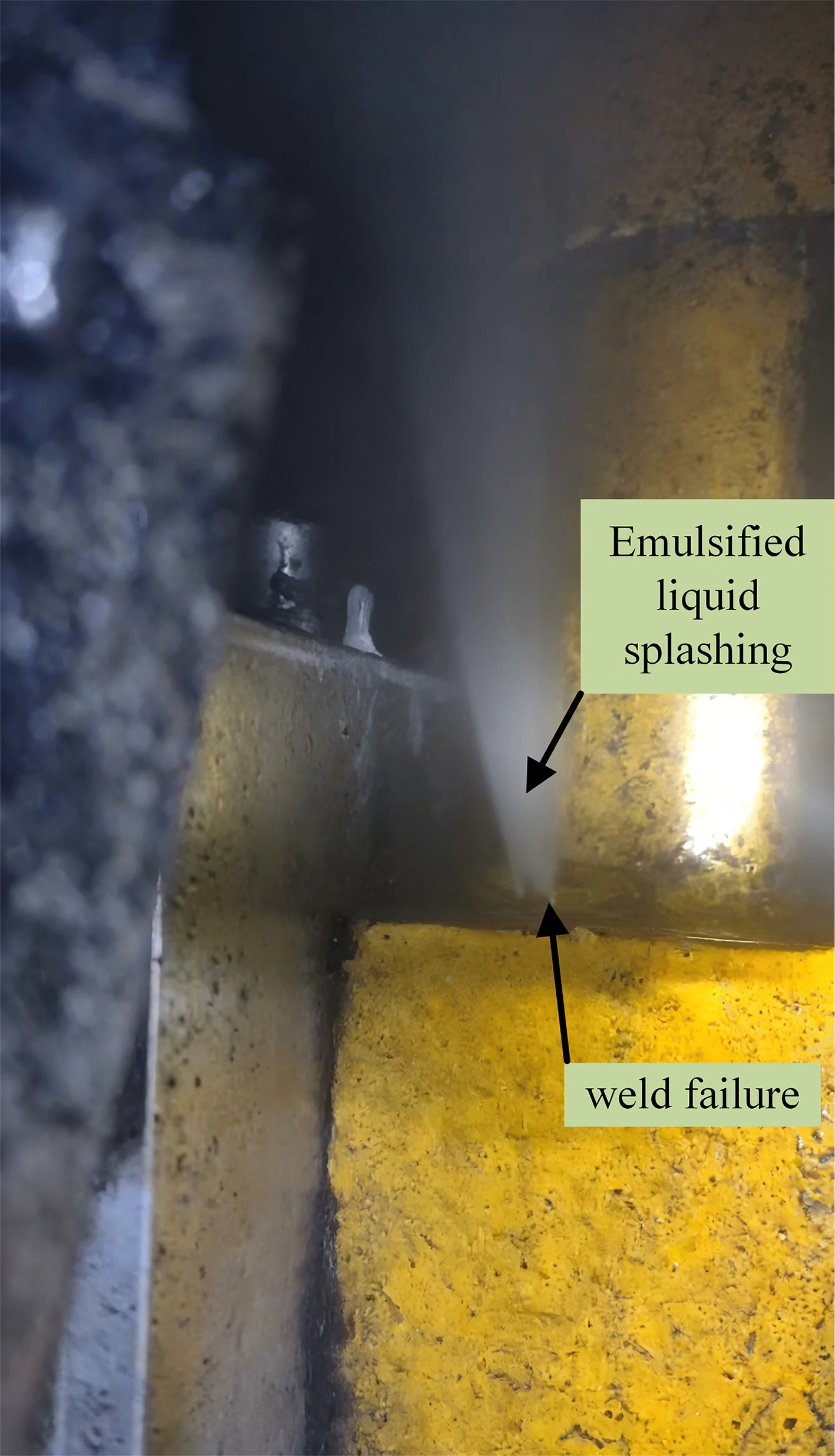
Cracking at the weld joint of the column outer weldment Image from Wang et al., 2025, distributed under a Creative Commons Attribution (CC BY 4.0) license.

Domestic and international scholars have carried out extensive research on hydraulic columns and achieved remarkable results. In terms of static and dynamic mechanical analysis of hydraulic columns, JianYing Liu et al. [25] established a numerical model for a prop with an outer diameter of φ320 mm, and numerically investigated the stress distributions under 1.5 times the rated axial load, an eccentric load of 30 mm, and 1.1 times the rated load. Liu Xinke et al. [26] studied the dynamic characteristics of props under impact loads, clarified the stress distribution law, and derived a theoretical calculation method for the maximum impact transmitted to the foundation. Most of the above studies adopt global response indicators such as maximum stress, impact force, displacement and deformation as evaluation criteria. Research concerning the axial spatial redistribution of stress within cylinder barrels, especially the correlation between local stress peaks near both end boundaries and the relatively steady stress state in the middle section, remains insufficient. In the field of structural improvement and anti-impact energy-absorbing structural design, Gamez-Montero P J et al. [27] explored the influence of friction torque at hinged joints on prop loading via experimental verification, and found that larger friction can improve axial mechanical conditions. Zhang Jianzhuo et al. [28] and An Dong et al. [29] investigated hydraulic shock issues and anti-impact performance of energy-absorbing anti-impact props, respectively, revealing the working mechanism of energy-absorbing components and the performance difference between energy-absorbing props and conventional props. Nevertheless, these studies mainly focus on impact attenuation. Few works reveal how the inherent geometric features of ordinary cylinder barrels govern the generation and severity of axial inhomogeneous stress distribution. In particular, the coupled effects of cylinder inner diameter, wall thickness and rodless chamber height on the stress difference between boundary regions and the middle barrel section have not been effectively analyzed. With regard to stress prediction and monitoring techniques, Zhang J. et al. [30] proposed a high-precision mapping method for prop stress by training data-driven models. Such approaches provide an efficient way to reconstruct the stress state of hydraulic columns, yet they fail to thoroughly investigate the stress characteristics of cylinder barrels and the underlying mechanical mechanisms responsible for stress redistribution.

Despite the progress achieved in existing studies, several issues related to the cylinder barrel itself remain insufficiently addressed. Most existing studies on impact dynamics concentrate on the overall response of hydraulic columns. Although previous literature has investigated stress concentration near the cylinder bottom and piston regions, few studies treat the axial inhomogeneity of the cylinder stress field as an independent mechanical characteristic. In particular, the coupling mechanism between this characteristic stress distribution and end constraints, cylinder geometry, as well as piston position has not been clearly clarified. Furthermore, most existing structural optimization studies aim to reduce the maximum stress and improve impact energy absorption capacity. Limited attention has been paid to the combined influences of cylinder inner diameter, wall thickness and rodless chamber height on the variation of peak-valley stress differences under combined static and dynamic loads.

To fill the above research gaps, this work focuses on the internal stress distribution of the cylinder barrel rather than the overall dynamic response of the whole prop. A mechanical model is established based on the stress law of thick-walled cylinders and the fundamental theory of rotating shells to describe the influence of end constraints on axial stress distribution. Subsequently, finite element simulations and full-scale static-dynamic loading tests are carried out to verify the stress characteristics predicted by the model. On this basis, the peak-valley stress differences on the inner and outer surfaces of the cylinder barrel under static and dynamic conditions are introduced as a quantitative index to evaluate the inhomogeneity of M-shaped stress distribution. Response surface methodology is adopted to analyze how this index varies with cylinder inner diameter, wall thickness and rodless chamber height.

The main contributions of this study are summarized in three aspects. First, the axial inhomogeneous stress distribution of the cylinder barrel is investigated from the perspective of coupling between thick-walled cylinder stress and boundary-induced end effects. The formation mechanism of two local stress peaks near the cylinder bottom and piston is explained from the mechanical viewpoint. Second, instead of merely adopting maximum stress as the evaluation criterion, the peak-valley stress differences on the inner and outer surfaces of the cylinder barrel under static and dynamic loads are utilized to characterize the M-shaped stress distribution. Third, a quantitative relationship between key geometric and operational parameters and the degree of stress inhomogeneity is established, which enables the identification of critical parameter combinations leading to deteriorated stress distribution in the cylinder barrel. The research results provide a reference for the design and working condition selection of anti-impact hydraulic columns and help avoid unfavorable structural configurations.

## 2. Mathematical model of stress distribution in cylinder barrel

To establish a mathematical model for the stress distribution of the hydraulic column and reveal its stress formation mechanism, the mechanical state of the cylinder barrel is analyzed by combining the thick-walled cylinder theory and the edge effect theory of rotating shells. According to the structural characteristics of the hydraulic column, the overall structure can be simplified into three parts: the end cap, the rodless chamber cylinder, and the rod chamber cylinder, as shown in Fig 2. The end cap is continuously connected to the cylinder bottom by welding. Considering that the end cap region possesses higher local constraint stiffness compared with the cylinder barrel, it is equivalently regarded as a rigid constraint zone to simplify the analytical solution.

**Fig 2:**
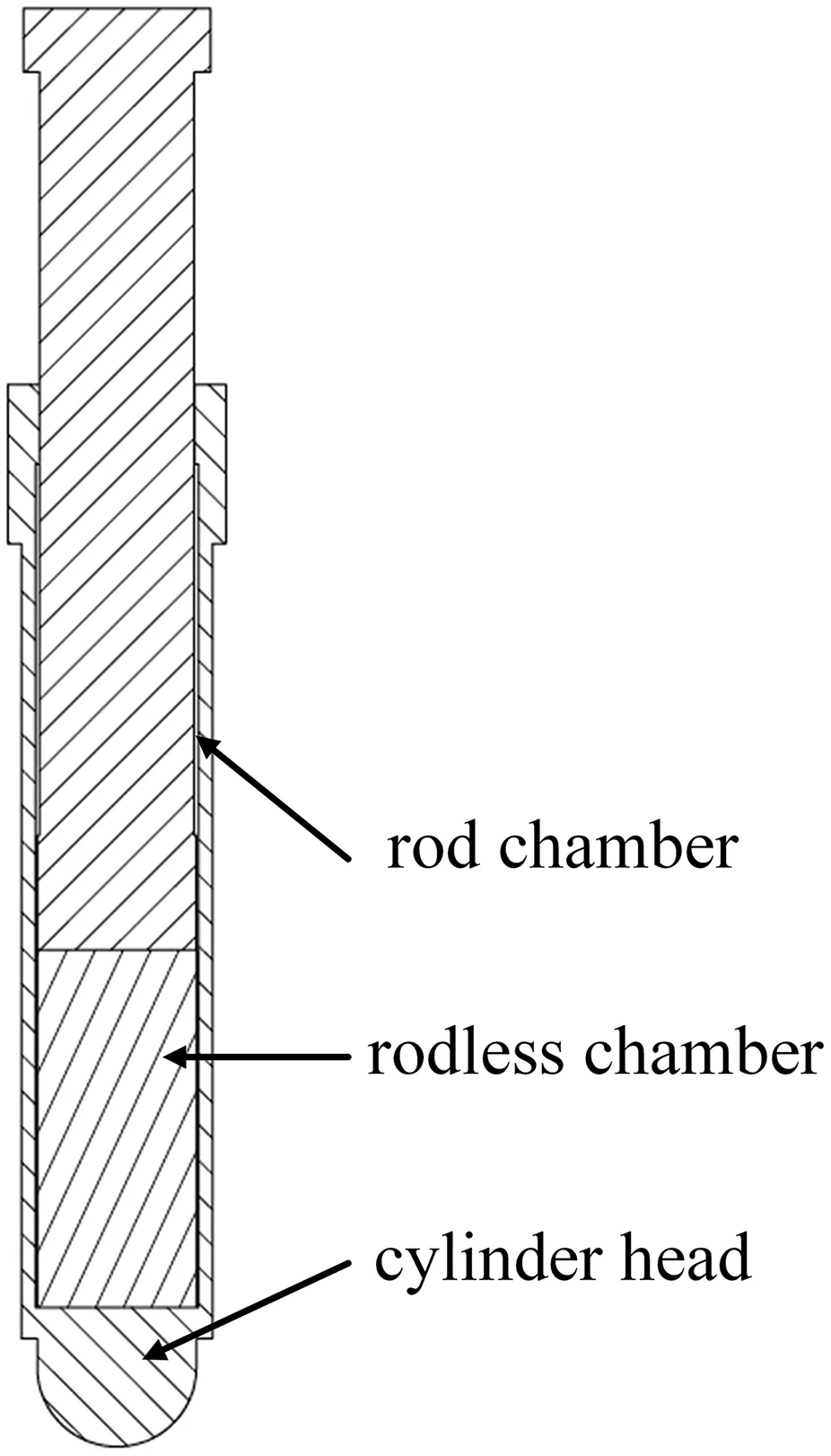
Simplified diagram of cylinder barrel.

For the cylinder regions far from the structural boundaries, the fundamental stress induced by internal hydraulic pressure can be accurately described by the classical thick-walled cylinder theory. However, near the structural boundaries of the cylinder bottom and piston side, the free radial expansion and rotation of the cylinder barrel under internal pressure are restricted due to the variations in geometric morphology and local constraint conditions. The unconstrained thick-walled cylinder solution cannot satisfy the continuity of radial displacement and rotation angle at the interface of adjacent structural regions, whereas the actual welded and contact connections require deformation compatibility between adjacent components.

To characterize such boundary constraint effects, equivalent edge forces and equivalent edge bending moments are introduced at the corresponding interfaces based on the edge effect theory of rotating shells, which can describe the additional local stress generated by interface deformation coordination. Consequently, the actual stress state of the hydraulic column is superposed by the fundamental stress of the thick-walled cylinder induced by internal hydraulic pressure and the additional edge stress caused by boundary constraints. On the premise of linear elasticity and small deformation assumptions, the two types of stress fields are combined via the superposition principle to establish the overall stress distribution model of the hydraulic column.

Based on the above mechanical decomposition strategy, the fundamental stress field of the cylinder barrel under internal hydraulic pressure is solved first. Subsequently, the equivalent edge forces and edge bending moments are determined according to the boundary deformation compatibility conditions, and the additional edge stress is further derived.

### 2.1 Stress of thick-walled cylinder

Under the axisymmetric internal pressure load, the radial stress and circumferential stress comply with the radial equilibrium equation. The inner surface of the cylinder barrel is subjected to hydraulic pressure P, while the outer surface is free of external loads. The corresponding boundary conditions are substituted into the general solution of the thick-walled cylinder theory, and the expressions of radial stress and circumferential stress shown in Equation (1) can be obtained [31]:


$$\begin{array}{c} \left\{ \begin{array}{c} @l@\sigma_{r}=(1-\frac{d_{\text{2}}^{\text{2}}}{r})Pd_{\text{1}}^{\text{2}}/\text{(}d_{\text{2}}^{\text{2}}-d_{\text{1}}^{\text{2}}\text{)}\\ \sigma_{\theta }=(1+\frac{d_{\text{2}}^{\text{2}}}{r})Pd_{\text{1}}^{\text{2}}/\text{(}d_{\text{2}}^{\text{2}}-d_{\text{1}}^{\text{2}}\text{)} \end{array}\right. \end{array}$$ (1)

Where, $\sigma_{r}$ is the radial stress, $\sigma_{\theta }$ is the hoop stress, *d*_1_ is the inner diameter of the cylinder barrel, *d*_2_ is the outer diameter of the cylinder barrel, *P* is the internal pressure on the inner wall of the cylinder barrel, *r* is the distance from the calculated node to the axis of the cylinder barrel.

### 2.2 Edge stress

Based on the equilibrium differential equations and geometric equations of shells, a preliminary analytical relationship between the internal forces and internal moments in the edge region is established. According to the requirements of geometric continuity and mechanical compatibility at the edge between the shell and the thick-walled cylinder, equations are derived under deformation compatibility conditions. The edge forces and edge moments are obtained through mathematical solution, and then the edge stresses of the cylinder barrel are determined.

The expression of the obtained edge stress is as follows:


$$\begin{array}{c} \left\{ \begin{array}{c} @l@\sigma_{x}=\frac{N_{x}^{\left(F+M\right)}}{S}\pm \frac{6M_{x}^{\left(F+M\right)}}{S^{\text{2}}}\\ {}[4pt]\sigma_{y}=\frac{N_{\theta }^{\left(F+M\right)}}{S}\pm \frac{6M_{\theta }^{\left(F+M\right)}}{S^{\text{2}}} \end{array}\right. \end{array}$$ (2)

Where, $\sigma_{x}$ is the axial stress, $\sigma_{y}$ is the hoop stress, $N_{x}^{\left(F+M\right)}$, $N_{\theta }^{\left(F+M\right)}$, $M_{x}^{\left(F+M\right)}$, $M_{\theta }^{\left(F+M\right)}$ are the internal force and internal moment generated by the edge force *F* and the edge bending moment *M*, *S* is the wall thickness of the cylinder barrel. Based on the obtained edge force and edge bending moment, the internal forces and internal moments are derived by combining the stress-internal force conversion relationship of rotating shells:


$$\begin{array}{c} \left\{ \begin{array}{c} @l@N_{x}^{\left(F+M\right)}=0\\ \left[4pt]N_{\theta }^{\left(F+M\right)}=e^{-kx}(2k^{\text{2}}rM\;cos\;kH-2krF\;cos\;kH-2k^{\text{2}}rM\;sin\;kH\right)\\ \left[4pt]M_{x}^{\left(F+M\right)}=e^{-kx}(M\;cos\;kH-\frac{F}{k}\;sin\;kH+M\;sin\;kH\right)\\ {}[4pt]M_{\theta }^{\left(F+M\right)}=\mu M_{x}^{\left(F+M\right)} \end{array}\right. \end{array}$$ (3)

Where, μ is the Poisson’s ratio of the material used for the cylinder barrel, and *H* is the distance from the calculated node to the edg, $k=\sqrt[{4}]{3(1-\mu^{\text{2}})}/\sqrt{rS}$. Under the action of internal moment, the edge of the cylinder barrel bends outward. The stress direction on the inner surface of the cylinder barrel is defined as the positive direction, and the outer surface as the negative direction. To realize deformation compatibility between adjacent structural regions, the interaction at each boundary is characterized by equivalent radial edge forces and equivalent edge bending moments. The physical quantities at the piston-side boundary of the rodless chamber are defined as P_0_ and M_0_, while those at the cylinder bottom boundary are defined as P_1_ and M_1_. These quantities are not externally applied loads, but internal reaction forces required to ensure the continuity of displacement and rotation angle between adjacent structural regions. Fig 3 shows the force diagram of the column cylinder barrel:

**Fig 3:**
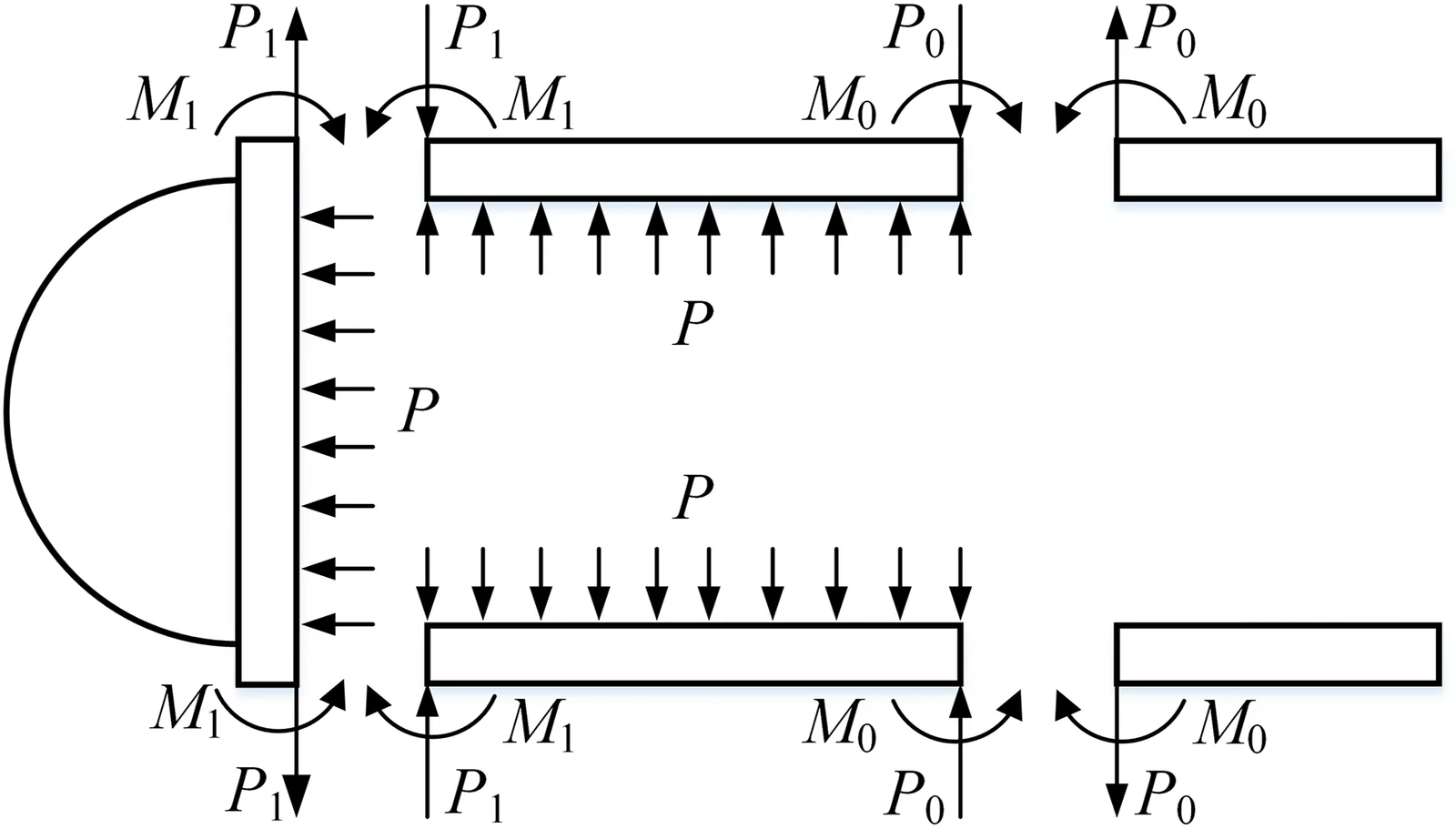
Schematic diagram of cylinder barrel edge coordination.

In the figure, for the convenience of analysis, the cylinder barrel is divided into three parts according to the head connection and the piston rod edge: the left head is Part 01, the middle rodless chamber section is Part 02, and the right rod chamber section is Part 03. *M*_0_ and *M*_1_ represent the edge moments on both sides of the rodless chamber of the cylinder barrel, *P*_0_ and *P*_1_ represent the corresponding edge forces, and *P* is the pressure of the emulsion inside the cylinder barrel.The deformation compatibility equations between Part 02 and Parts 01, 03 are as follows:


$$\begin{array}{c} \left\{ \begin{array}{c} @l@\Delta_{01}^{\left(P\right)}+\Delta_{01}^{P_{\text{0}}}+\Delta_{01}^{M_{\text{0}}}=\Delta_{02}^{\left(P\right)}+\Delta_{02}^{P_{\text{0}}}+\Delta_{02}^{M_{\text{0}}}\\ {}[4pt]\theta_{01}^{\left(P\right)}+\theta_{01}^{P_{\text{0}}}+\theta_{01}^{M_{\text{0}}}=\theta_{02}^{\left(P\right)}+\theta_{02}^{P_{\text{0}}}+\theta_{02}^{M_{\text{0}}} \end{array}\right. \end{array}$$ (4)

$$\begin{array}{c} \left\{ \begin{array}{c} @l@\Delta_{02}^{\left(P\right)}+\Delta_{02}^{P_{\text{1}}}+\Delta_{02}^{M_{\text{1}}}=\Delta_{03}^{\left(P\right)}+\Delta_{03}^{P_{\text{1}}}+\Delta_{03}^{M_{\text{1}}}\\ {}[4pt]\theta_{02}^{\left(P\right)}+\theta_{02}^{P_{\text{1}}}+\theta_{02}^{M_{\text{1}}}=\theta_{03}^{\left(P\right)}+\theta_{03}^{P_{\text{1}}}+\theta_{03}^{M_{\text{1}}} \end{array}\right. \end{array}$$ (5)

Where, $\Delta^{\left(P\right)}$is the deformation of the cylinder barrel caused by the internal wall pressure, $\Delta^{P_{\text{0}}}$ and $\Delta^{P_{\text{1}}}$are the deformations induced by the edge forces, $\Delta^{M_{\text{0}}}$and$\Delta^{M_{\text{1}}}$ are the deformations caused by the edge bending moments. The inward radial direction of the edge circle is defined as the positive direction of Δ. $\theta^{\left(P\right)}$is the edge rotation angle of the cylinder barrel due to the internal wall pressure, $\theta^{M_{\text{0}}}$and $\theta^{M_{\text{1}}}$are the edge rotation angles caused by the edge forces, and and are the edge rotation angles induced by the edge bending moments. The clockwise direction is defined as the positive direction of θ.The deformations and edge rotation angles of the cylinder and circular plate under edge forces and edge bending can be expressed respectively as [32]:


$$\begin{array}{c} \left\{ \begin{array}{c} @l@\Delta_{\text{T}}^{\left(P\right)}=-\frac{Pr^{\text{2}}}{2ES_{\text{1}}}(2-\mu ),\theta_{\text{T}}^{\left(P\right)}=0\\ {}[4pt]\Delta_{\text{T}}^{P1}=\frac{P_{\text{0}}}{2k_{\text{1}}^{\text{3}}D_{\text{1}}},\theta_{\text{T}}^{P1}=-\frac{P_{\text{0}}}{2k_{\text{1}}^{\text{2}}D_{\text{1}}}\\ {}[4pt]\Delta_{\text{T}}^{M1}=\frac{M_{\text{0}}}{2k_{\text{1}}^{\text{2}}D_{\text{1}}},\theta_{\text{T}}^{M1}=-\frac{M_{\text{0}}}{k_{\text{1}}D_{\text{1}}} \end{array}\right.\text{\textbackslash{}textcircled\{1}\},\left\{ \begin{array}{c} @l@\Delta_{\text{B}}^{\left(P\right)}=0,\theta_{\text{B}}^{\left(P\right)}=\frac{Pr^{\text{3}}}{8(1+\mu )D_{\text{2}}}\\ {}[4pt]\Delta_{\text{B}}^{P}=\frac{(1-\mu )rP_{\text{0}}}{ES_{\text{2}}},\theta_{\text{B}}^{P}=-\frac{P_{\text{0}}S_{\text{2}}r}{2(1+\mu )D_{\text{2}}}\\ {}[4pt]\Delta_{\text{B}}^{M}=0,\theta_{\text{B}}^{M}=\frac{rM_{\text{0}}}{(1+\mu )D_{\text{2}}} \end{array}\right.\text{②} \end{array}$$ (6)

Where, $\Delta_{\text{T}}$is the deformation of the cylinder barrel, $\Delta_{\text{B}}$is the deflection of the end‑cap circular plate, $\theta_{\text{T}}$is the edge rotation of the cylinder barrel, $\theta_{\text{B}}$is the edge rotation of the end‑cap circular plate, *E* is the elastic modulus, *S*_1_ is the wall thickness of the cylinder barrel, and *S*_2_ is the wall thickness of the end‑cap circular plate， $k_{\text{1}}=\sqrt[{4}]{3(1-\mu^{\text{2}})}/\sqrt{rS_{\text{1}}}$, $D_{\text{1}}=ES_{\text{1}}^{\text{3}}/12(1-\mu^{\text{2}})$, $D_{\text{2}}=ES_{\text{2}}^{\text{3}}/12(1-\mu^{\text{2}})$.

By judging the directions of deformation and edge rotation between Part 02 and Part 03, and substituting Equation (6)① into the corresponding deformation compatibility Equation (5), we can obtain:


$$\begin{array}{c} \left\{ \begin{array}{c} @l@-\frac{Pr^{\text{2}}}{2ES_{\text{1}}}(2-\mu )+\frac{P_{\text{0}}}{2k_{\text{1}}^{\text{3}}D_{\text{1}}}+\frac{M_{\text{0}}}{2k_{\text{1}}^{\text{2}}D_{\text{1}}}=-\frac{P_{\text{0}}}{2k_{\text{1}}^{\text{3}}D_{\text{1}}}+\frac{M_{\text{0}}}{2k_{\text{1}}^{\text{2}}D_{\text{1}}}\\ {}[6pt]\frac{P_{\text{0}}}{2k_{\text{1}}^{\text{2}}D_{\text{1}}}+\frac{M_{\text{0}}}{k_{\text{1}}D_{\text{1}}}=\frac{P_{\text{0}}}{2k_{\text{1}}^{\text{2}}D_{\text{1}}}-\frac{M_{\text{0}}}{k_{\text{1}}D_{\text{1}}} \end{array}\right. \end{array}$$ (7)

By substituting *k*_1_ and *D*_1_ into Equation (9) respectively, the edge forces *P*_0_ and edge moments *M*_0_ of Part 02 and Part 03 can be obtained as:


$$\begin{array}{c} \left\{ \begin{array}{c} @l@P_{\text{0}}=\frac{Pr^{\text{2}}k_{\text{1}}^{\text{3}}S_{\text{1}}^{\text{2}}}{36(1-\mu^{\text{2}})}(2-\mu )\\ \left[6pt]M_{\text{0}}=\frac{Pr^{\text{2}}k_{\text{1}}^{\text{2}}S_{\text{1}}^{\text{2}}}{-72(1-\mu^{\text{2}})}(2-\mu \right) \end{array}\right. \end{array}$$ (8)

Substituting Equation (8) into Equation (2), the stress at the right edge of Part 02 is solved as:


$$\begin{array}{c} \left\{ \begin{array}{c} \sigma_{x}^{\text{0}}=\pm \frac{\text{6}}{S_{\text{1}}^{\text{2}}}e^{-k_{\text{1}}H}[M_{\text{0}}\;cos(k_{\text{1}}H)+(M_{\text{0}}-\frac{P_{\text{0}}}{k_{\text{1}}})sin\;\left(k_{\text{1}}H\right)]\\ {}[5pt]\sigma_{y}^{\text{0}}=(e^{-k_{\text{1}}H}[(2k_{\text{1}}^{\text{2}}rM_{\text{0}}-2k_{\text{1}}rP)\;cos\;\left(k_{\text{1}}H\right)-2k_{\text{1}}^{\text{2}}rM_{\text{0}}\;sin\;\left(k_{\text{1}}H\right)]/S_{\text{1}})+\mu \sigma_{x}^{\text{0}} \end{array}\right. \end{array}$$ (9)

Restricted by the functional requirements of energy absorption, liquid supply, and installation, hydraulic columns are equipped with various forms of end caps. Taking the end cap of the unit hydraulic column in this study as an example, the composite structure consisting of a hemisphere and a circular plate makes it difficult to accurately solve the internal forces and moments induced by P_1_ and M_1_. Mechanical analysis indicates that the emulsion pressure P acting on the right side of the end cap counteracts the supporting force on the left side. Thus, the influence of hydraulic pressure P on the edge force and edge moment of the cylinder junction can be neglected. For simplification, the end cap is equivalent to a circular plate without hydraulic pressure on its right side. Numerical simulation is conducted to verify the feasibility of this simplification. Fig 4 compares the inner and outer wall stress of the cylinder barrel between the original end cap model and the circular plate simplified model with different wall thicknesses under a loading pressure of 10 MPa.

**Fig 4:**
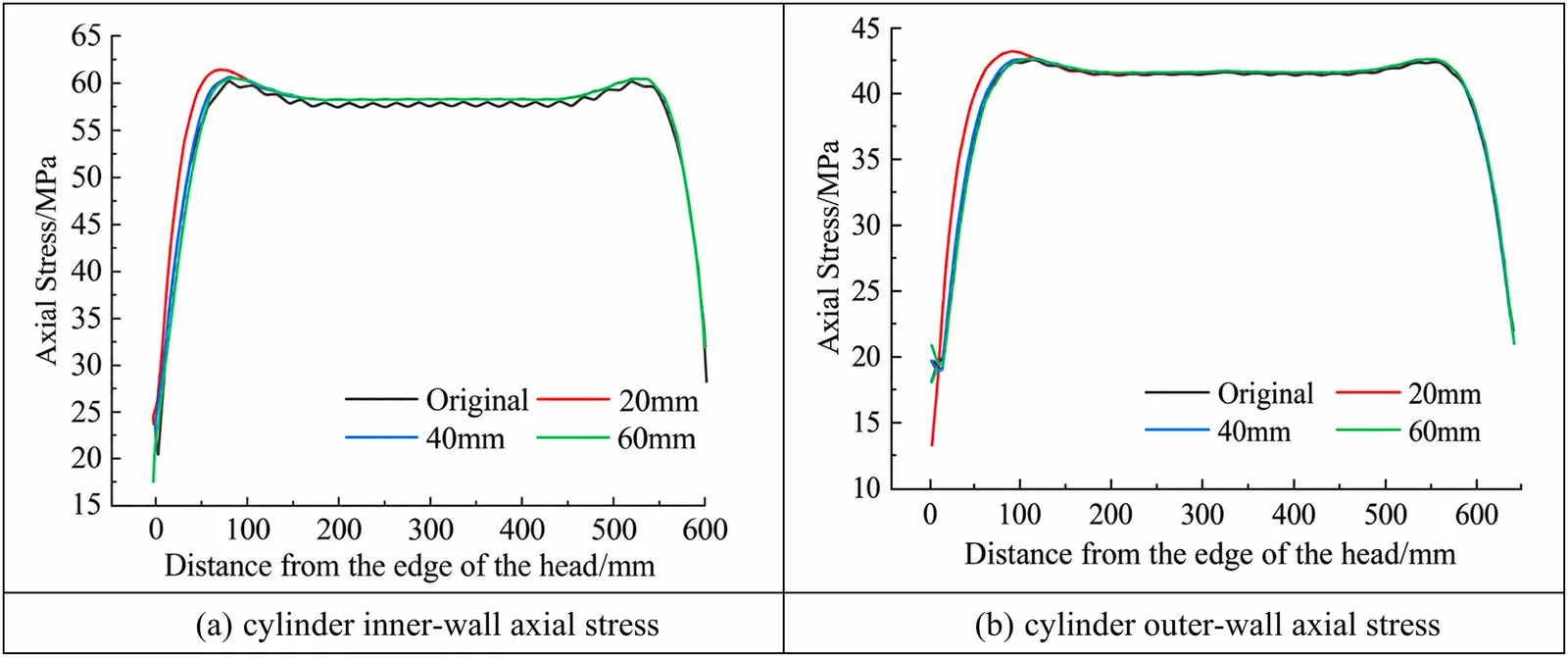
Comparison of stresses between the realistic model and the simplified model.

As illustrated in Fig 4, the stress distributions of the circular plate simplified models with different wall thicknesses are highly consistent with those of the original model. The results verify that the proposed simplification has negligible influence on the solution accuracy. Therefore, the simplified model does not affect the solution accuracy. Substituting Equation (6)② into Equation (4), the deformation compatibility equations at the edge between Part 01 and Part 02 can be obtained as:


$$\begin{array}{c} \left\{ \begin{array}{c} @l@-\frac{Pr^{\text{2}}}{2ES_{\text{1}}}(2-\mu )+\frac{P_{\text{1}}}{2k_{\text{1}}^{\text{3}}D_{\text{1}}}+\frac{M_{\text{1}}}{2k_{\text{1}}^{\text{2}}D_{\text{1}}}=\frac{-(1-\mu )P_{\text{1}}r}{ES_{\text{2}}}+\frac{S_{\text{2}}}{\text{2}}(\frac{-P_{\text{1}}S_{\text{2}}r}{2(1+\mu )D_{\text{2}}}+\frac{M_{\text{1}}r}{(1+\mu )D_{\text{2}}})\\ {}[6pt]\frac{-P_{\text{1}}}{2k_{\text{1}}^{\text{2}}D_{\text{1}}}-\frac{M_{\text{1}}}{k_{\text{1}}D_{\text{1}}}=\frac{-P_{\text{1}}S_{\text{2}}r}{2(1+\mu )D_{\text{2}}}+\frac{M_{\text{1}}r}{(1+\mu )D_{\text{2}}} \end{array}\right. \end{array}$$ (10)

Substituting *k*_1_, *D*_1_, and *D*_2_ into Equation (10) and setting μ=0.3, the edge force *P*_1_ and edge moment *M*_1_ between Part 02 and Part 01 can be obtained respectively as:


$$\begin{array}{c} \left\{ \begin{array}{c} @l@k_{\text{1}}M_{\text{1}}=\frac{[16.1\frac{S_{\text{2}}}{r}-12.67(\frac{S_{\text{2}}}{S_{\text{1}}}{)}^{\text{3}}\frac{S_{\text{1}}}{r}]Pr}{38.32(\frac{S_{\text{2}}}{S_{\text{1}}}{)}^{\text{3}}\sqrt{\frac{S_{\text{1}}}{r}}+83.52(\frac{S_{\text{2}}}{S_{\text{1}}}{)}^{\text{2}}\frac{S_{\text{1}}}{r}+97.4\frac{S_{\text{2}}}{S_{\text{1}}}\sqrt{\frac{S_{\text{1}}}{r}}+144.48\frac{S_{\text{1}}}{S_{\text{2}}}\sqrt{\frac{S_{\text{1}}}{r}}+75.78}\\ {}[12pt]P_{\text{1}}=\frac{[13.879+14.0366(\frac{S_{\text{2}}}{S_{\text{1}}}{)}^{\text{3}}\sqrt{\frac{S_{\text{1}}}{r}}]k_{\text{1}}M_{\text{1}}}{18.92\frac{S_{\text{2}}}{S_{\text{1}}}\sqrt{\frac{S_{\text{1}}}{r}}-7.018(\frac{S_{\text{2}}}{S_{\text{1}}}{)}^{\text{3}}\sqrt{\frac{S_{\text{1}}}{r}}} \end{array}\right. \end{array}$$ (11)

Similarly, substituting Equation (11) into Equation (2), the stress at the left edge of Part 02 is solved as:


$$\begin{array}{c} \left\{ \begin{array}{c} \sigma_{x}^{\text{1}}=\pm \frac{\text{6}}{S_{\text{1}}^{\text{2}}}e^{-k_{\text{1}}H}[M_{\text{1}}cos(k_{\text{1}}H)+(M_{\text{1}}-\frac{P_{\text{1}}}{k_{\text{1}}})sin\left(k_{\text{1}}H\right)]\\ {}[6pt]\sigma_{y}^{\text{1}}=(e^{-k_{\text{1}}H}[(2k_{\text{1}}^{\text{2}}rM_{\text{1}}-2k_{\text{1}}rP)cos\left(k_{\text{1}}H\right)-2k_{\text{1}}^{\text{2}}rM_{\text{1}}sin\left(k_{\text{1}}H\right)]/S_{\text{1}})+\mu \sigma_{x}^{\text{1}}array \end{array}\right. \end{array}$$ (12)

After obtaining the stresses of the thick-walled cylinder and the edge stresses respectively, the stress of the column cylinder barrel can be expressed as:


$$\begin{array}{c} \sigma =\frac{\text{1}}{\sqrt{\text{2}}}\sqrt{(\sigma_{\text{1}}-\sigma_{\text{2}}{)}^{\text{2}}+(\sigma_{\text{2}}-\sigma_{\text{3}}{)}^{\text{2}}+(\sigma_{\text{3}}-\sigma_{\text{1}}{)}^{\text{2}}} \end{array}$$ (13)

The above-obtained thick-walled cylinder stress $\sigma_{r}\text{,}\sigma_{\theta }$ and edge stress $\sigma_{x}^{\text{0}}$, $\sigma_{y}^{\text{0}}$, $\sigma_{x}^{\text{1}}$, $\sigma_{y}^{\text{1}}$ are both principal stresses, where $\sigma_{\text{1}}=\sigma_{\theta }+\sigma_{y}^{\text{0}}+\sigma_{y}^{\text{1}}$, $\sigma_{\text{2}}=\sigma_{x}^{\text{0}}+\sigma_{x}^{\text{1}}$, $\sigma_{\text{3}}=\sigma_{r}$. Based on the above mechanism model, the main factors influencing the stress distribution of the hydraulic column cylinder barrel are the cylinder diameter *d*_1_, cylinder wall thickness *S*, rodless chamber height *x* of the column, and emulsion pressure *P* inside the cylinder barrel. To investigate the influence characteristics of each factor on the stress distribution of the cylinder barrel, the stress distribution law of the hydraulic column cylinder barrel is explored via finite element simulation analysis.

## 3. Investigation of stress distribution characteristics of cylinder barrel

### 3.1 Development of finite element model for cylinder barrel

In this study, the LD20191000 type hydraulic column is taken as the research object, with its main structural parameters as follows: inner diameter of 200 mm, wall thickness of 21.5 mm, and stroke of 1000 mm. A three-dimensional model was established using SolidWorks software and then imported into ANSYS Workbench for finite element analysis. The key parameter settings in the finite element modeling are specified as follows: the height of the rodless chamber inside the cylinder barrel is 900 mm; the working medium inside the cylinder barrel is emulsion; the material of the column cylinder barrel is 35CrMnSiA, with a density of 7850 kg/m³, an elastic modulus of 210 GPa, and a Poisson’s ratio of 0.3.

Constraints are applied on the lower supporting surface of the hydraulic column to replicate the supporting conditions in the full-scale loading test and eliminate rigid body motion. The external axial load is applied on the upper end face of the piston rod along the prop axis, consistent with the primary load transmission direction of the hydraulic column. The remaining constraints are only used to suppress rigid body motion without imposing restrictions on the radial deformation of the cylinder barrel. The simplified model is shown in Fig 5, which mainly consists of the cylinder barrel, piston rod and emulsion.

**Fig 5:**
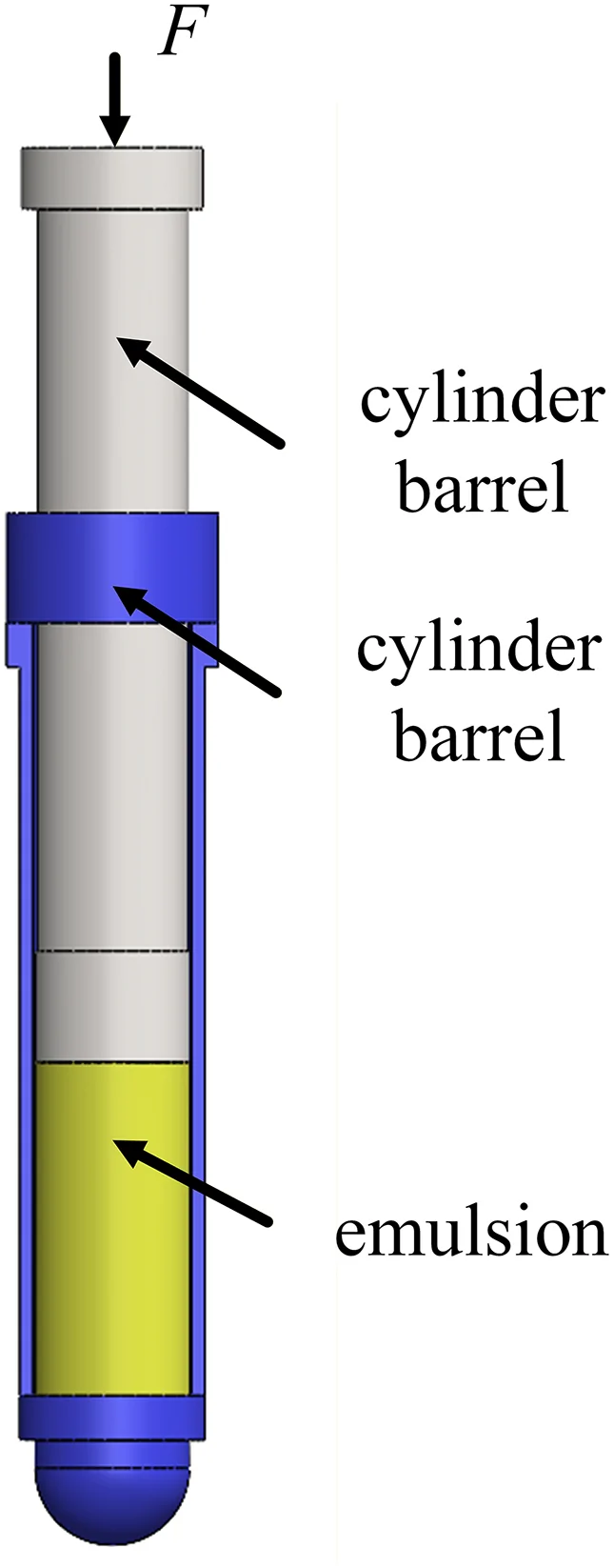
Simplified simulation model.

### 3.2 Numerical simulation

Numerical simulations are carried out on the simplified model. The static structural analysis is performed using the Static Structural module. The end time of the analysis step is set to 1 s with one step, and automatic time stepping is controlled by the solver.

The transient dynamic response of the hydraulic column was investigated using LS-DYNA. The reference model had a cylinder inner diameter of 200 mm, a wall thickness of 21.5 mm, and a rodless-chamber height of 900 mm. Based on the rated working condition of the investigated hydraulic column, the peak axial dynamic load was specified as 1256 kN，load is applied using a ramp–time curve. The rise time and loading duration were 15 ms and 15 ms, respectively, with the peak load reached at 15ms.

The total simulation duration was 30 ms, selected based on the actual loading duration and the time range required to capture the dominant transient stress responses. The transient analysis employed the explicit time-integration scheme. For the explicit analysis, the stable time increment was automatically controlled by LS-DYNA according to the element characteristic length and material wave speed. The time-step safety factor was 0.9. In the finite-element analysis, the axial normal stress component, *σ*_z_, was extracted along the inner and outer surfaces of the cylinder barrel using paths parallel to the cylinder axis. The axial coordinate z was measured from the cylinder-bottom reference position. The corresponding axial stress distributions were used to investigate the formation and variation of the M-shaped stress profile under static and dynamic loading. Calculation results are exported at 200 equally spaced sampling points.

To quantify the axial stress non-uniformity, four response variables were defined: Δ*σ*_so_ and Δ*σ*_si_ for the outer and inner surfaces under static loading, and Δ*σ*_do_ and Δ*σ*_di_ for the outer and inner surfaces under dynamic loading, respectively. All four responses were calculated from the peak-to-valley differences of the same axial stress component, *σ*_z_.

To evaluate the reliability of the transient simulation results, the numerical load time history was further compared with the experimental loading conditions to clarify their correspondence in terms of peak load, loading duration and stages of dynamic response. Given the simplifications adopted in the transient model and the idealized loading assumptions, the numerical model is primarily used to analyse the cylinder stress distribution and local dynamic amplification characteristics under specified impact conditions.

The piston rod is defined as a rigid body, while the cylinder barrel and emulsion are defined as deformable bodies. This treatment is adopted because this work focuses on the internal stress redistribution within the cylinder barrel. The deformation of the piston rod only exerts a secondary influence on the cylinder responses of interest, and this simplification can also reduce the computational cost of transient analysis. Frictional contact is defined between the piston rod and cylinder barrel with a friction coefficient of 0.15. Hexahedral meshes are adopted for the cylinder barrel, piston rod and emulsion, with an element size of 5 mm.

To evaluate the mesh dependence of stress results, a mesh convergence analysis was implemented prior to formal numerical simulations. Five sets of hexahedral element sizes (3 mm, 5 mm, 10 mm, 15 mm, and 20 mm) were adopted, while material properties, contact definitions, boundary conditions and loading conditions remained unchanged. For each mesh model, stress values at identical stress peak locations on the inner and outer surfaces of the cylinder barrel under static and dynamic loading were extracted along the same axial path, and the results are listed in the table.

As shown in Table 1, the calculated stress peaks gradually converge with mesh refinement. The stress peaks change obviously when the element size decreases from 20 mm to 5 mm, while the variation becomes much smaller as the element size is below 5 mm. When the mesh size is further refined from 5 mm to 3 mm, the relative variations of static outer-wall stress peak, static inner-wall stress peak, dynamic outer-wall stress peak and dynamic inner-wall stress peak are 0.035%, 0.03%, 0.37% and 0.514%, respectively, all less than 1%, which meets the requirement of numerical calculation accuracy.

**Table 1:** Stress values at the identical peak‑stress position of the cylinder barrel under different element sizes.

Nominal element size (mm)	Static outer‑surface peak stress (MPa)	Static inner‑surface peak stress (MPa)	Transient outer‑surface peak stress (MPa)	Transient inner‑surface peak stress (MPa)
3	171.83	238.86	376.42	482.92
5	171.77	238.79	375.03	480.45
10	170.65	237.43	364.15	465.64
15	169.20	236.97	358.64	455.51
20	168.48	235.67	345.19	440.97

The above results demonstrate that further mesh refinement based on the 5 mm mesh has limited influence on the predicted stress peaks. Considering both numerical accuracy and computational efficiency, a mesh size of 5 mm is adopted for the subsequent static, transient and response surface simulations.

As analyzed from the mathematical model of the stress distribution of the column cylinder barrel, the main factors affecting the stress distribution state of the column cylinder barrel include the height of the rodless chamber of the column, the cylinder barrel diameter, the cylinder barrel wall thickness, and the emulsion pressure inside the cylinder barrel. The emulsion pressure was set to 40 MPa for analysis.

For the static structural analysis, a constant pressure of 40 MPa was applied to the inner surface of the cylinder barrel, and the results of the static numerical simulation are shown in Fig 6(a); in the transient dynamic analysis, an impact load of 1.256 × 10^6^ N is applied on the top of the piston rod, corresponding to an emulsion pressure of 40 MPa inside the cylinder barrel, and the corresponding transient dynamic simulation results are presented in Fig 6(b).

**Fig 6:**
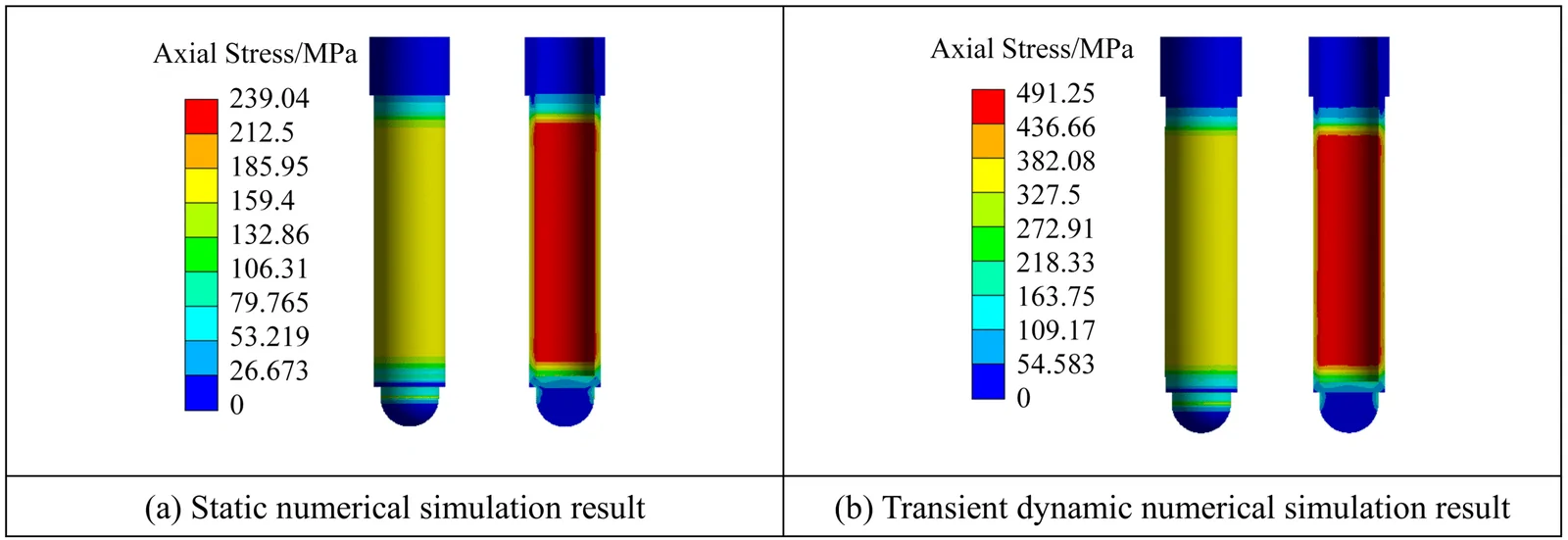
Numerical simulation results of cylinder barrel.

Since the cylinder barrel is cylindrical, the inner and outer walls of the cylinder barrel were selected to draw radial PATH curves for analyzing the stress distribution. A total of 200 nodes were set for both the inner and outer wall PATH curves, and the stress distribution along these curves is shown in Fig 7.

**Fig 7:**
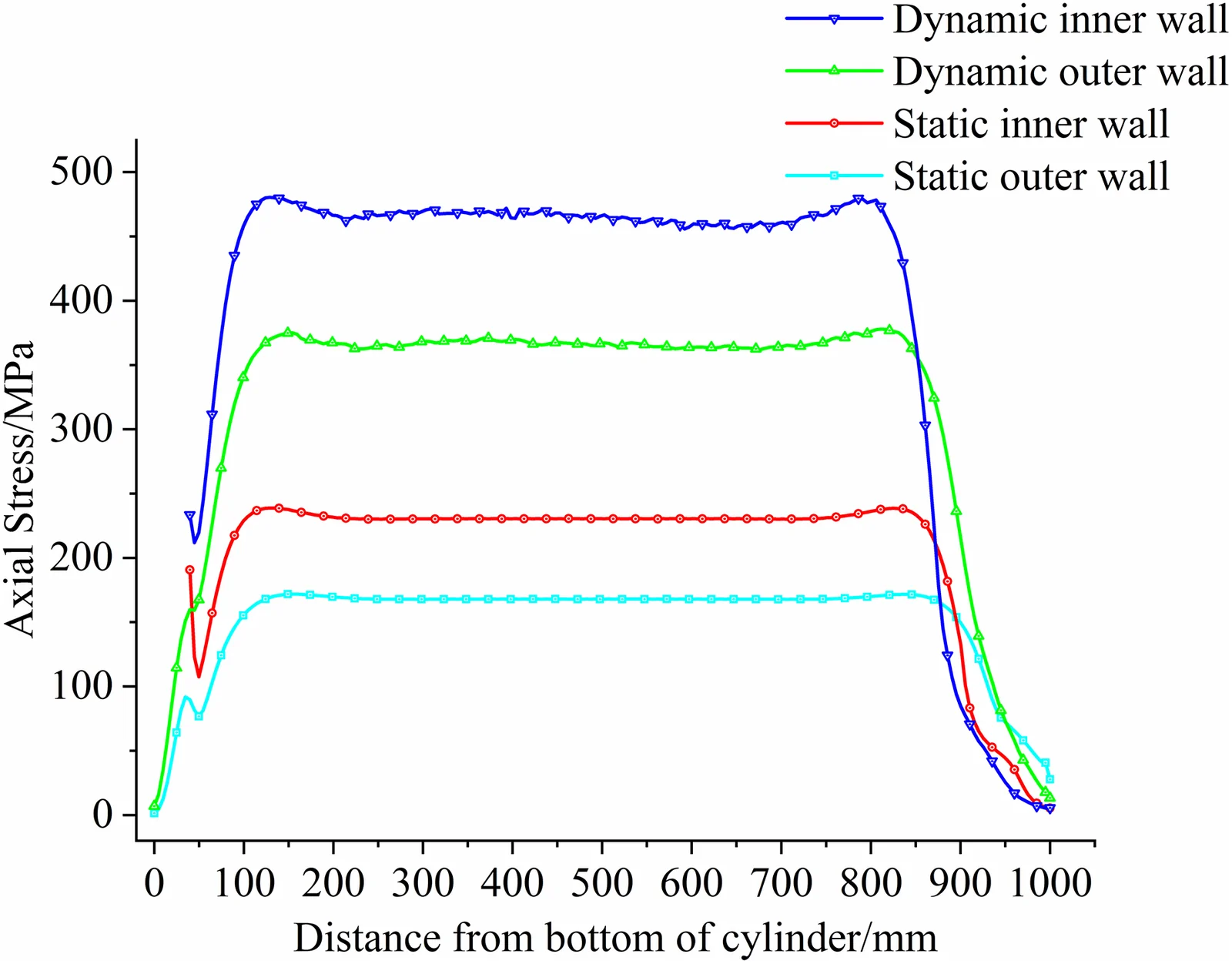
Axial Stress curve of cylinder barrel.

As shown in the figure, the axial stress value gradually increases with the increase of the distance between the node position and the bottom of the cylinder barrel; all four curves exhibit stress fluctuations at approximately 40 mm from the bottom of the cylinder barrel, which is the joint between the head and the cylinder barrel. At about 140 mm from the bottom end of the cylinder barrel, the static outer wall axial stress reaches a peak value of 171.77 MPa, the static inner wall axial stress reaches a peak value of 238.79 MPa, the dynamic outer wall axial stress reaches a peak value of 375.03 MPa, and the dynamic inner wall axial stress reaches a peak value of 480.45 MPa. At approximately 830 mm from the bottom end of the cylinder barrel, all four curves reach the second peak, with values of 171.65 MPa (static outer wall), 238.53 MPa (static inner wall), 377.86 MPa (dynamic outer wall), and 479.46 MPa (dynamic inner wall) respectively. Within the range of 190 mm to 750 mm from the bottom of the cylinder barrel, the axial stress values of the four curves tend to stabilize. Under both static and dynamic load conditions, the stress curves of the inner and outer walls generally show an “M” shape, with the two stress peaks located near the bottom of the cylinder barrel and the piston rod, i.e., the cusp positions of the “M” shape. The difference (Δσ_so_) between the maximum static outer wall stress and the minimum stress in the middle of the rodless chamber is 4.03 MPa; the difference (Δσ_si_) between the maximum static inner wall stress and the minimum stress in the middle of the rodless chamber is 8.7 MPa; the difference (Δσ_do_) between the maximum dynamic outer wall stress and the minimum stress in the middle of the rodless chamber is 15.82 MPa; and the difference (Δσ_di_) between the maximum dynamic inner wall stress and the minimum stress in the middle of the rodless chamber is 24.7 MPa.

The larger the difference between the maximum stress on the inner and outer walls of the cylinder barrel and the minimum stress in the middle of the rodless chamber, the more pronounced the “M”-shaped characteristic of the stress curve becomes. The area near the stress peak of the cylinder barrel is subjected to more severe loading conditions, and the cylinder barrel is more prone to swelling failure. Therefore, researching the “M”-shaped characteristic of the stress curve is of great significance for the safe operation of hydraulic columns.

## 4. Experimental research

### 4.1 Experimental equipment

The 6500 kN static-dynamic composite loading test machine [33] was adopted to conduct tests on the hydraulic column. The specifications of the column are as follows: inner diameter of 200 mm, wall thickness of 21.5 mm, total stroke of 1000 mm, rated working pressure of 40 MPa, and rated working resistance of 1.256 × 10^6^ N. The 6500 kN static-dynamic composite loading test machine is capable of performing loading test research on hydraulic columns, and its main structure consists of three parts: a measurement and control system, a test bench, and a hydraulic loading system, as shown in Fig 8.

**Fig 8:**
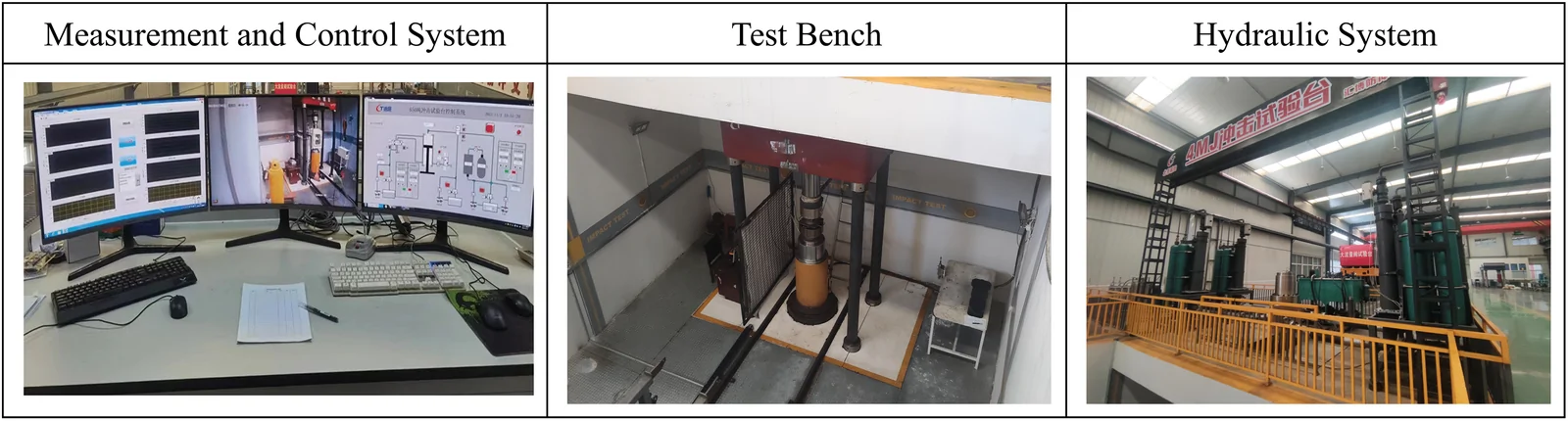
6500kN static and dynamic composite loading testing machine Image from Wang et al., 2025, distributed under a Creative Commons Attribution (CC BY 4.0) license.

### 4.2 Experimental plan and results

The stress state of the hydraulic column cylinder barrel was mainly monitored by strain gauges. The distribution of the strain gauges is specified as follows: a total of 12 layers of strain gauges were arranged along the radial direction of the cylinder barrel from top to bottom, with 3 columns in each layer, resulting in 36 strain gauges in total. Strain gauges were bonded along the axial direction of the cylinder barrel to measure axial strain. Uniaxial foil-type resistance strain gauges were adopted in the test, with a gauge factor of 2.0 and measurement accuracy of 1%–2%. The data sampling frequency was set to 20000 Hz. Since the ambient temperature fluctuated slightly during the test, no additional temperature compensation gauge was arranged. Noise in the acquired signals was suppressed following the hierarchical strategy: hardware noise suppression first, then digital filtering, and finally post-processing of raw data. The measured strain was converted into stress via Equation (14).


$$\begin{array}{c} \sigma_{D}=E\varepsilon_{D} \end{array}$$ (14)

Where *ε*_D_ and *σ*_D_ denote the experimentally measured axial strain and the corresponding calculated axial stress, respectively.

The strain gauges in each layer were placed at intervals of 120°, 60°, and 60° in the clockwise direction. The physical diagram of the column and the strain gauges is shown in Fig 9:

**Fig 9:**
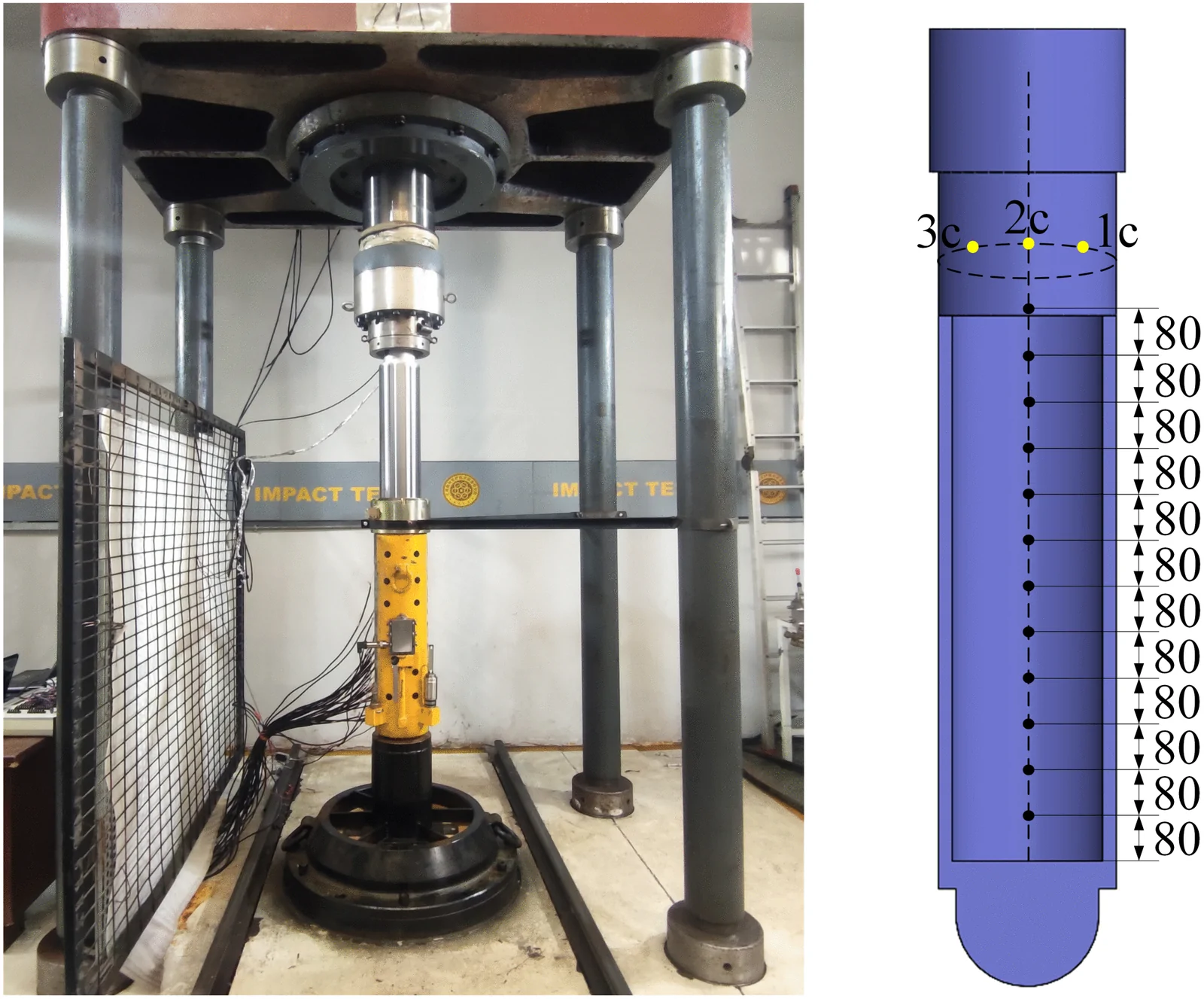
Physical diagram of hydraulic column and strain gauge Image from Wang et al., 2026, distributed under a Creative Commons Attribution (CC BY 4.0) license.

For the loading test of the hydraulic column, the column was placed vertically on the ground. Static load tests and dynamic impact tests were mainly conducted, with the applied loads of 1.056 × 10^6^ N, 1.256 × 10^6^ N, 1.456 × 10^6^ N, and 1.656 × 10^6^ N respectively. The height of the emulsion in the hydraulic column was 900 mm. After converting the test data from strain to stress and summarizing, the results are shown in Fig 10.

**Fig 10:**
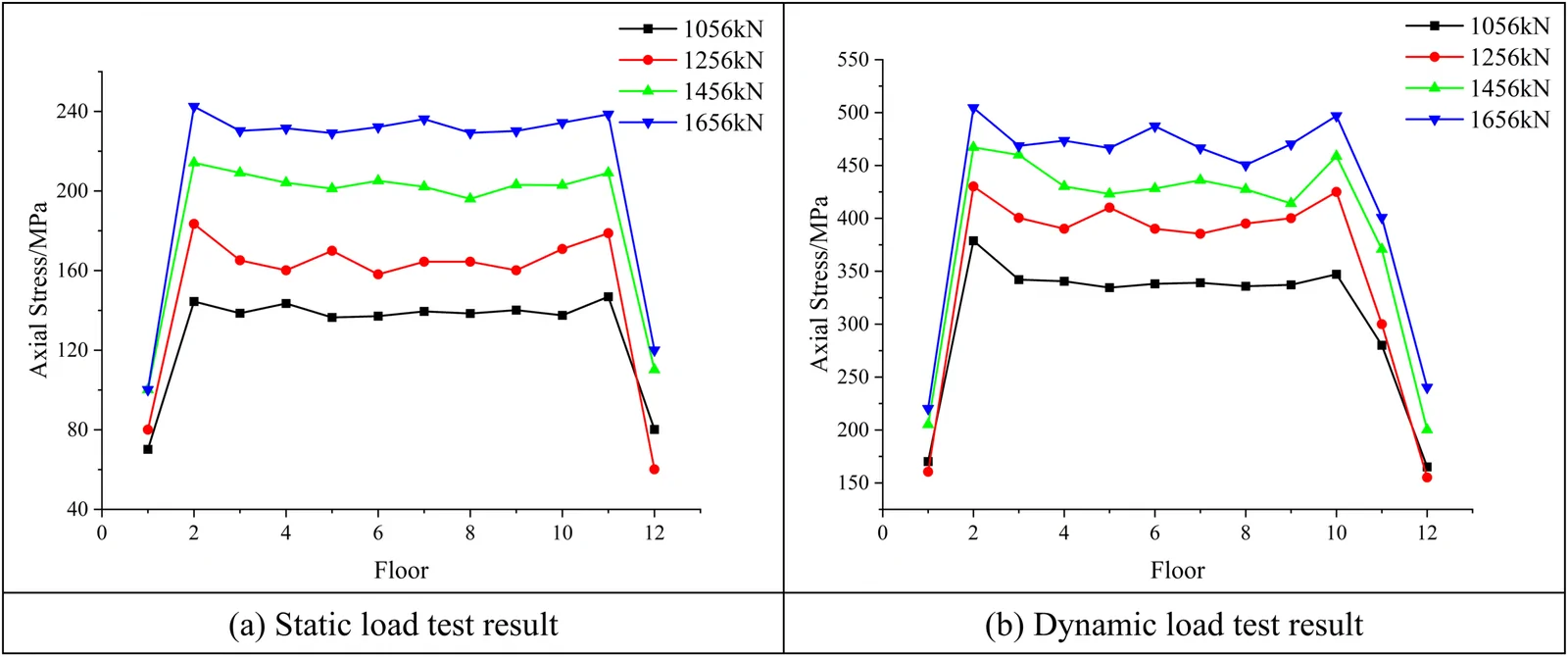
Test data of hydraulic column.

Under different applied load conditions, the experimental research and numerical simulation results of the column cylinder barrel show that the stress curves all present an “M”-shaped trend, indicating a high degree of consistency between the experimental results and the numerical simulation. Specifically, under static load, the stress variation in the middle of the rodless chamber of the cylinder barrel is relatively gentle, while significant stress peaks are observed near the head and the piston rod. This phenomenon can be attributed to the geometric characteristics of the cylinder barrel structure and the inhomogeneity of the load distribution.

A further comparison of the experimental results under dynamic load reveals that the stress variation in the middle of the rodless chamber of the cylinder barrel is more intense than that under static load. This is mainly due to the dynamic load being affected by multiple factors such as vibration effects and inertial effects, leading to complex changes in the internal stress state of the cylinder barrel. Although the maximum positions of the stress peaks under dynamic load are similar to those under static load—both located near the head and the piston rod—the stress fluctuations and peak intensity induced by dynamic load are significantly increased. This finding is of great significance for understanding the mechanical properties of the cylinder barrel under complex working conditions, and provides a theoretical basis for the optimal design and safety assessment of the cylinder barrel structure.

### 4.3 Error analysis between experimental results and numerical simulation

Fig 11 compares the experimental results with the finite element simulation results of the cylinder barrel. Since it is not feasible to measure the inner wall stress of the cylinder barrel in the experiment, only the outer wall stress is compared.

**Fig 11:**
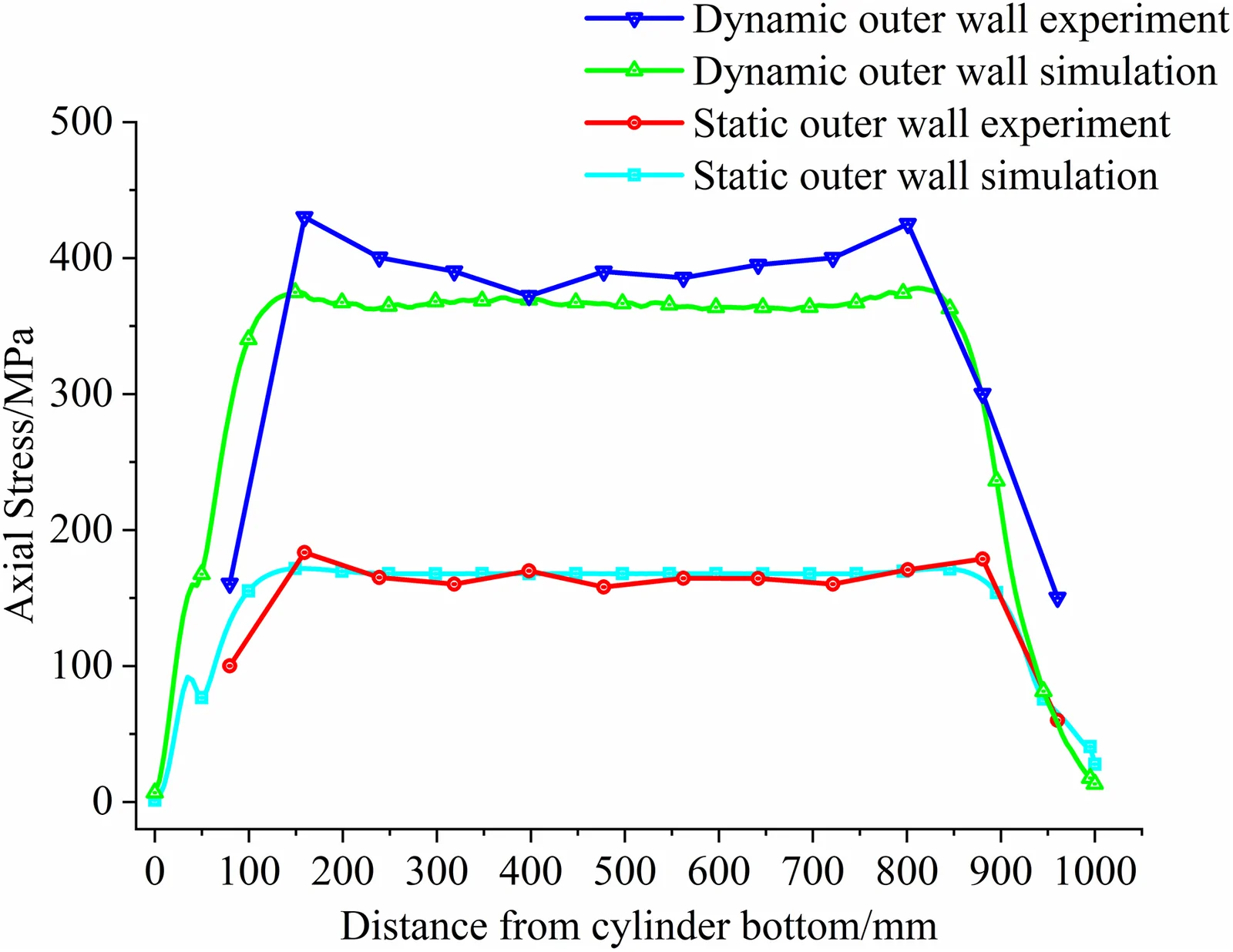
Comparison between finite element numerical simulation and test results.

Under static and dynamic loads, the variation trends of experimental results are basically consistent with those obtained from finite element numerical simulations. The stress changes slightly in the middle of the rodless chamber of the cylinder barrel, and stress peaks appear near the end cap and piston. The stress nodes obtained from experiments are compared point-by-point with the corresponding nodes in the numerical simulation results, and the comparison results are listed in Table 2. It can be seen from Table 2 that under static loading, the maximum error between numerical simulation and experiment is within 8%, and the average error is less than 4%. Under dynamic loading, the maximum error is within 15% and the average error is below 10%. This indicates that the established numerical model has satisfactory accuracy and can reasonably reflect the stress distribution state of the cylinder barrel under practical working conditions.

**Table 2:** Error comparison between finite element numerical simulation and test results.

	Max. Error	Min. Error	Avg. Error
Error under static load	7.97%	1.03%	3.60%
Error under dynamic load	14.95%	5.83%	9.45%

Although the stress distribution trends obtained from experimental tests and numerical simulations are consistent, minor discrepancies still exist due to measurement errors and modeling simplifications. Therefore, this section systematically analyzes the uncertainty sources in both experimental measurements and numerical simulations to clarify the error mechanisms and verify the model reliability.

For experimental testing, the main uncertainties originate from strain gauge accuracy, installation deviation, and stress conversion errors. In this study, the cylinder stress is converted from measured strain data based on the elastic constitutive relationship. Thus, the inherent errors and discreteness of material elastic parameters directly introduce deviations to the calculated stress results. Meanwhile, the curved surface of the cylinder barrel, as well as the positioning accuracy and bonding quality of strain gauges, cause local measurement errors and further affect the test accuracy. In addition, pressure fluctuations of the hydraulic loading system lead to dynamic deviations in applied load, introducing additional experimental uncertainty.

For numerical simulation, uncertainties mainly arise from material assumptions, simplified boundary conditions, contact parameter settings, and equivalent treatment of dynamic loads. The cylinder material is assumed to be homogeneous and linearly elastic in the numerical model, whereas the mechanical properties of actual engineering materials exhibit certain discreteness and nonlinear characteristics. Furthermore, the fixed constraints and frictional contact conditions adopted in the finite element model are idealized approximations of practical service conditions. The actual dynamic impact process is simplified as equivalent impact load input, which differs from the complex real loading process and induces numerical calculation errors.

Comprehensive analysis of various uncertainty factors indicates that the discrepancies between numerical predictions and experimental measurements are within a reasonable range. The average errors under static and dynamic loading conditions are 3.60% and 9.45%, respectively, demonstrating acceptable numerical accuracy. The established finite element model is reliable and can accurately reproduce the axial stress distribution and mechanical response characteristics of hydraulic columns, which satisfies the requirements for subsequent parametric analysis and mechanical mechanism investigation.

## 5. Exploration on the influence characteristics of stress distribution in cylinder barrel

Based on the analysis of the mathematical model and finite element model for the stress distribution of the column cylinder barrel, combined with the investigation of the column loading test, it is verified that the stress of the cylinder barrel presents an “M”-shaped distribution regardless of whether it is subjected to static or dynamic loads, with stress peaks existing near the head and the piston. When the cylinder barrel stress shows an “M”-shaped distribution, the larger the difference between the maximum stress and the minimum stress in the middle of the rodless chamber, the more uneven the force distribution in the middle of the rodless chamber becomes, and the more prone the cylinder barrel is to swelling failure. To study the variation law of this stress difference when the cylinder barrel stress exhibits an “M”-shaped distribution, the response surface methodology (RSM) was adopted to analyze the influence characteristics of the stress distribution of the column cylinder barrel.

### 5.1 Response surface methodology (RSM) experimental design

As indicated by the mathematical model for the stress distribution of the column cylinder barrel, the main factors affecting the stress distribution of the column cylinder barrel are the cylinder inner diameter, cylinder wall thickness, and the height of the rodless chamber inside the cylinder barrel (the distance from the calculated node to the bottom of the cylinder barrel). The response surface methodology (RSM) was adopted, simulation tests are carried out using Ansys Workbench. The cylinder bore *d*_1_, cylinder wall thickness *S*, and rodless-chamber height *H* are selected as experimental factors. For all cases, the static load corresponds to a pressure of 40 MPa, while the dynamic load adopts the working resistance of each cylinder bore under 40 MPa. The factor levels are specified in Table 3.

**Table 3:** Factors and levels of response surface experiment.

Influencing factors	Symbols	Levels		
		−1	0	1
Cylinder barrel inner diameter /mm	*d*_1_	200	215	230
Cylinder barrel wall thickness /mm	*S*	20	21.5	23
Height of rodless chamber in cylinder barrel /mm	*H*	100	500	900

The value ranges of the three influencing factors are comprehensively determined based on the prototype structural dimensions, the mechanical characteristics derived from the numerical model, and the actual working interval of hydraulic columns. The established parameter ranges constitute a locally effective design domain with practical engineering significance for the research objects in this study, rather than universal dimensional limits applicable to all types of hydraulic columns.

The LD20191000 hydraulic column is adopted as the reference structure, with a prototype inner diameter of 200 mm, a cylinder wall thickness of 21.5 mm, and a total working stroke of 1000 mm. Under the working pressure of 40 MPa, the theoretical supporting force is approximately 1.256 × 106 N at an inner diameter of 200 mm and increases to 1.662 × 106 N at an inner diameter of 230 mm. This diameter range achieves an effective variation in bearing capacity and conforms to the practical engineering application of the same type of hydraulic columns. The wall thickness varies from 20 mm to 23 mm, floating up and down based on the practical thickness of 21.5 mm. Within the matched range of inner diameter and wall thickness adopted in this study, the thickness-to-diameter ratio is maintained between 0.174 and 0.230, which satisfies the thick-walled cylinder assumption of the analytical model. The rodless chamber height is set from 100 mm to 900 mm, corresponding to 10%–90% of the total stroke of the reference prop. The two mechanical limit positions are excluded to eliminate excessive interference induced by extreme displacement constraints and contact effects. Variation in the rodless chamber height adjusts the axial distance between the boundary influence zones near the cylinder bottom and piston, which enables the capture of the evolution law of M-shaped stress distribution under diverse working conditions. In summary, the three-factor variable domain adopted in the Box–Behnken design (BBD) represents the practical engineering design and working interval of the studied hydraulic columns.

A test design table generated by the Box-Behnken design (BBD) method [34–37] was adopted, and simulations were conducted using ANSYS Workbench software. Taking the cylinder inner diameter (*d*_*1*_), cylinder wall thickness (*S*), and rodless chamber height (*H*) as the test factors, A three-factor, three-level test was carried out, consisting of 17 test groups in total.

In this study, the stress peak-to-valley difference (Δ*σ*) is used to quantify the spatial non-uniformity of the specified stress component along the effective rodless-chamber region of the cylinder barrel. For an identifiable M-shaped stress profile, Δ*σ* is determined from the selected local stress peak(s) and the intermediate stress valley according to the prescribed extraction procedure. The four response variables, Δ*σ*_so_, Δ*σ*_si_, Δ*σ*_do_, and Δ*σ*_di_, correspond to the static outer-wall, static inner-wall, dynamic outer-wall, and dynamic inner-wall responses, respectively. The same stress-component definition and peak–valley identification rules are applied consistently to the corresponding numerical results.

Among them, 5 test groups were repeated tests with the same parameters to handle test errors. The test results are shown in Table 4.

**Table 4:** Response surface experimental plan and results.

Std	Run	*d*_*1*_	*S*	*H*	Δ*σ*_*so*_	Δ*σ*_*si*_	Δ*σ*_*do*_	Δ*σ*_*di*_
8	1	230	21.5	900	4.92	10.67	53.97	68.52
2	2	230	20	500	5.45	11.55	44.58	61.43
5	3	200	21.5	100	0.00	0	0	0
1	4	200	20	500	4.56	9.72	33.39	52.51
7	5	200	21.5	900	4.03	8.70	51.20	64.64
11	6	215	20	900	4.84	10.47	62.70	71.69
12	7	215	23	900	4.15	8.94	52.81	70.48
9	8	215	20	100	0	0	0	0
16	9	215	21.5	500	4.64	9.74	38.55	58.66
10	10	215	23	100	0	0	0	0
13	11	215	21.5	500	4.66	9.70	38.20	57.95
15	12	215	21.5	500	4.58	9.87	39.16	59.28
14	13	215	21.5	500	4.70	9.66	38.49	58.23
4	14	230	23	500	4.66	10.13	37.48	57.76
6	15	230	21.5	100	0	0	0	0
17	16	215	21.5	500	4.55	9.80	39.65	59.65
3	17	200	23	500	3.86	8.19	32.49	54.17

### 5.2 Establishment of response value prediction model

Based on the values of R^2^ (coefficient of determination), Adjusted R^2^ (adjusted coefficient of determination), and Predicted R^2^ (predicted coefficient of determination) corresponding to the quadratic polynomial prediction models for each response value in the response surface experiment [38–41], the Quadratic model was selected as the prediction model type for all four response values, namely the stress differences between the stress peaks (on the inner and outer walls of the cylinder barrel) and the stress at the cavity under both static and dynamic conditions. Statistical information of the model is presented in Table 5. Residual analysis was conducted based on these prediction models. Fig 12, Fig 13, Fig 14 present the normal probability plots of the internally studentized residuals, the plots of the internally studentized residuals versus the predicted values, and the plots of the predicted values versus the actual values for each response value.

**Table 5:** Statistical fitting of the prediction model.

Response	R^2^	Adjusted R^2^	Predicted R^2^	Adeq Precision	Std.Dev.	Mean	C.V.%
Δ*σ*_so_	0.9974	0.9940	0.9610	44.1686	0.1581	3.51	4.51
Δ*σ*_si_	0.9977	0.9948	0.9650	47.4294	0.3128	7.48	4.18
Δ*σ*_do_	0.9944	0.9872	0.9134	36.0549	2.32	33.10	7.01
Δ*σ*_di_	0.9982	0.9959	0.9734	54.1991	1.75	46.76	3.75

**Fig 12:**
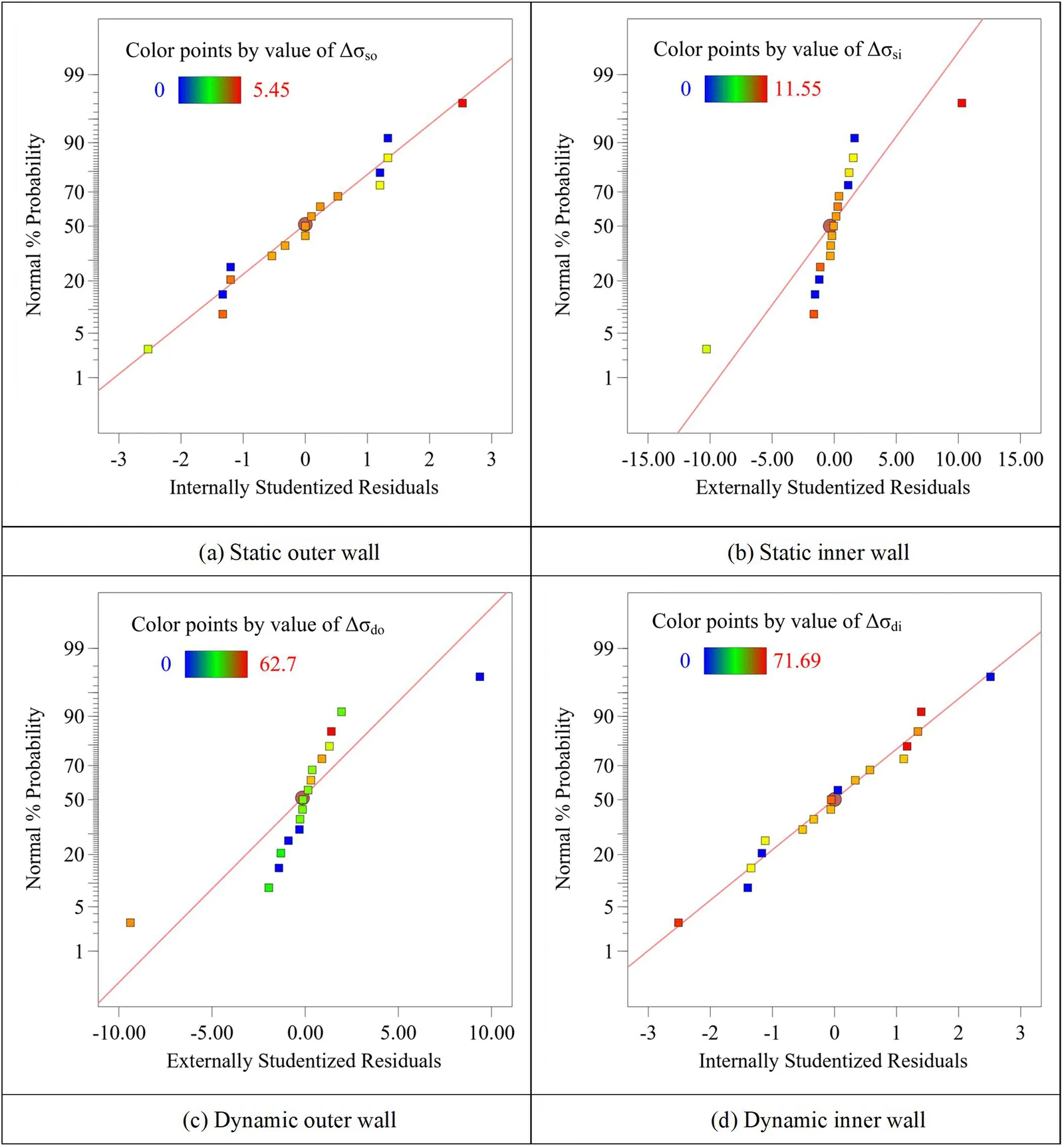
Normal distribution probability diagrams of the internally studentized residuals.

**Fig 13:**
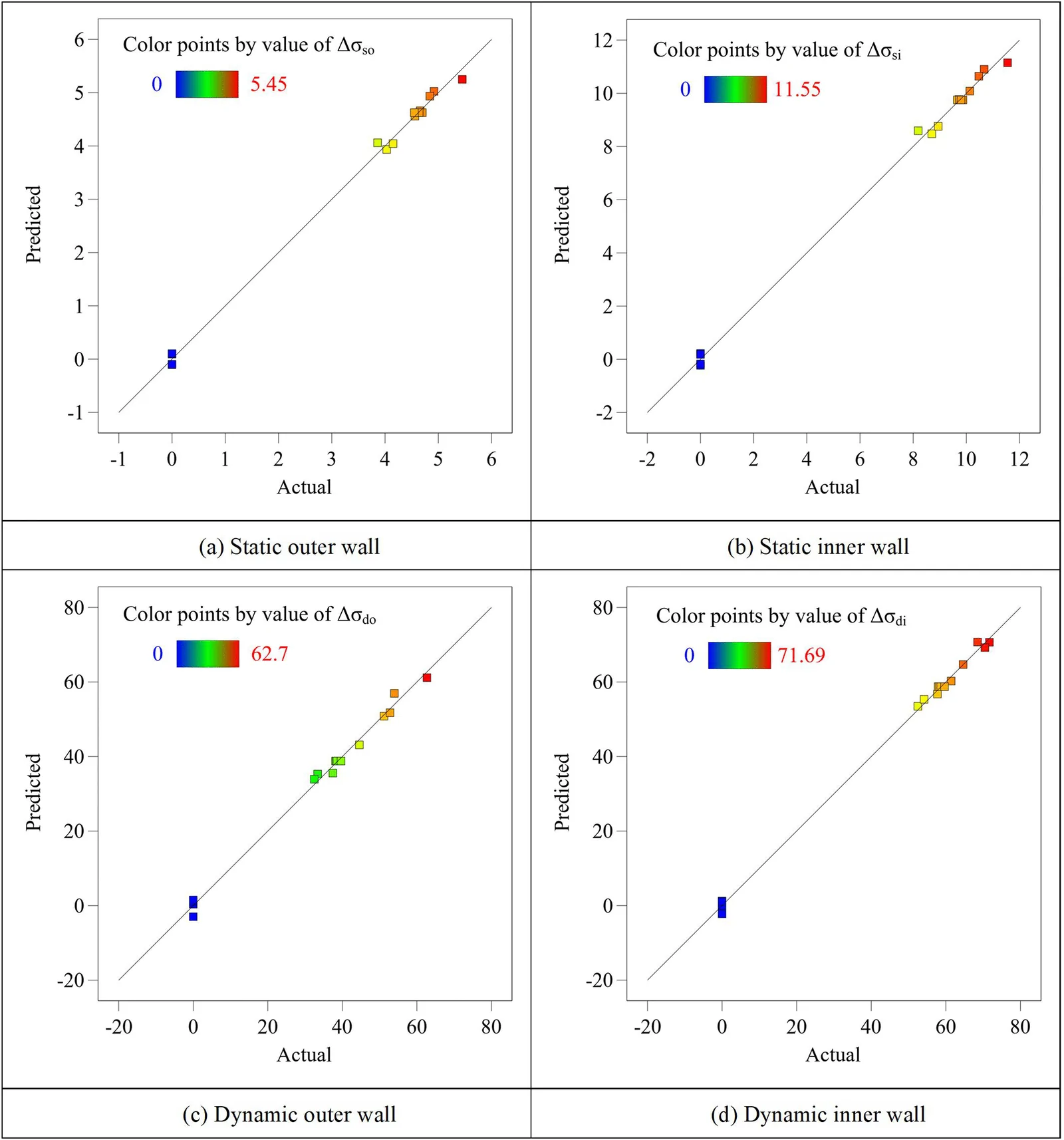
Distribution diagrams of the predicted values and the actual values.

**Fig 14:**
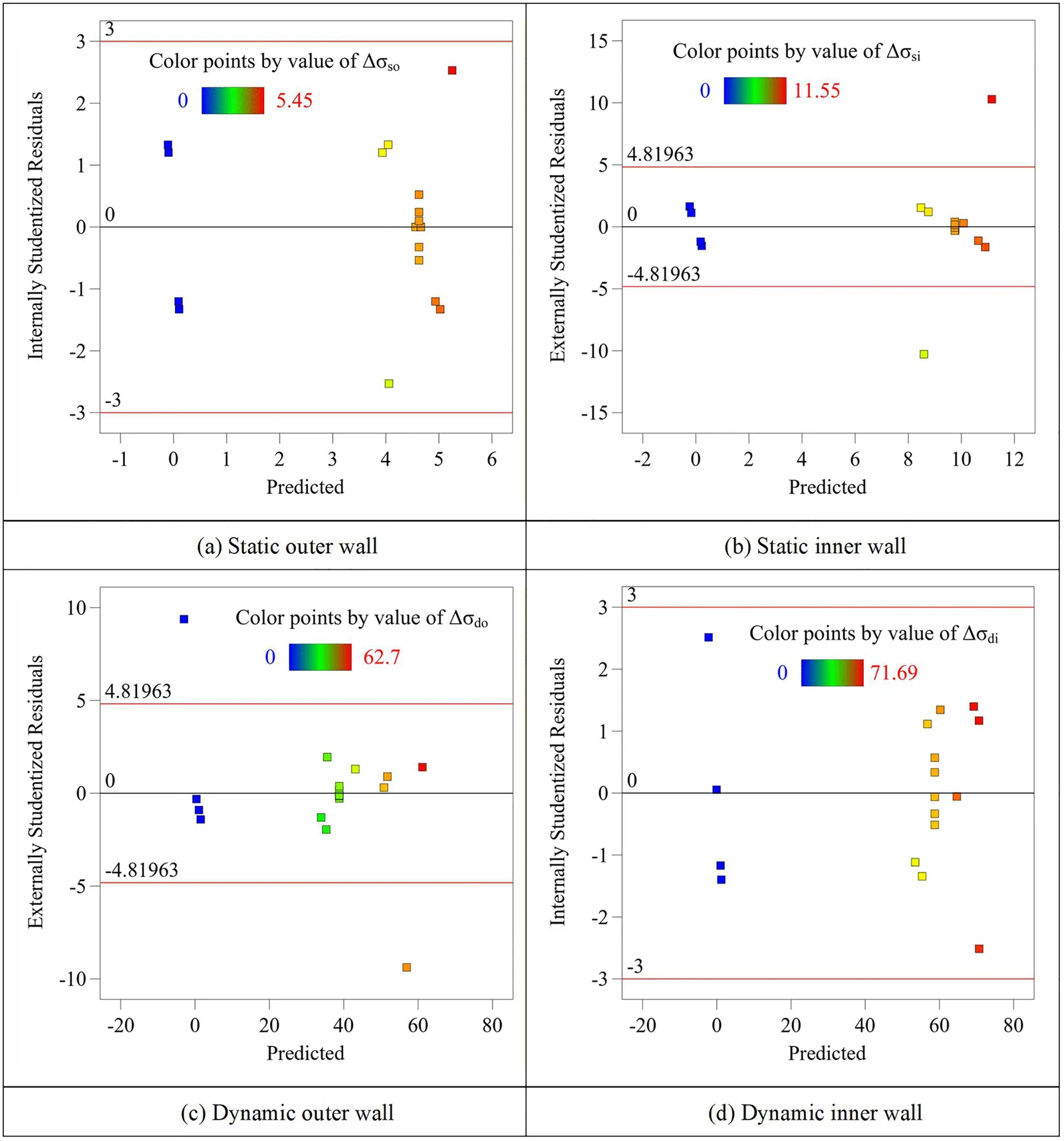
Distribution diagrams of the internally studentized residuals and the predicted values.

#### 5.2.1 Static stress difference of cylinder barrel outer wall Δ*σ*_*so*_.

For the prediction model of the static outer-wall stress peak-to-valley difference (Δσ_so_), the coefficient of determination (R^2^), adjusted R^2^, and predicted R^2^ are 0.9974, 0.9940, and 0.9610, respectively, indicating that the quadratic model captures a substantial proportion of the variation in the FE-derived responses. The relatively small difference between the adjusted and predicted R² values suggests reasonable internal predictive consistency. The coefficient of variation (C.V.) is 4.51%, while the adequate precision reaches 32.3241, exceeding the recommended threshold of 4 and indicating an adequate signal for exploring the investigated design space. Nevertheless, the lack-of-fit test is statistically significant (F = 14.51, p = 0.0129), revealing systematic deviations that are not fully captured by the quadratic approximation. Therefore, despite its high overall fitting performance, the model should be regarded as an approximate representation of the numerical response rather than a fully adequate predictive model.

Significance test and analysis of variance (ANOVA) were performed on the prediction model of Δ*σ*_so_. The results are shown in Table 6. The results show that the model’s F-value reaches 293.89 with a P-value less than 0.0001, indicating high statistical significance of the model. The P-values of the cylinder inner diameter (*d*_1_), wall thickness (*S*), rodless chamber height (*H*), interaction term *d*_1_*H*, interaction term *SH*, and quadratic term *H*^2^ are all less than 0.05, demonstrating that each term has a significant effect on the response value.

**Table 6:** ANOVA for Quadratic model of the Δ*σ*_so._

Source	Sum of Squares	df	Mean Square	F-value	P-value	
Model	66.10	9	7.34	293.89	<0.0001	significant
*d*_1_-*d*_1_	0.8320	1	0.8320	33.30	0.0007	
*S*-*S*	0.5940	1	0.5940	23.77	0.0018	
*H*-*H*	40.23	1	40.23	1609.95	<0.0001	
*d*_1_ *S*	0.0020	1	0.0020	0.0810	0.7841	
*d*_1_ *H*	0.1980	1	0.1980	7.92	0.0260	
*SH*	0.1190	1	0.1190	4.76	0.0654	
*d*_1_^2^	0.0000	1	0.0000	0.0005	0.9825	
*S* ^2^	0.0003	1	0.0003	0.0115	0.9177	
*H* ^2^	23.99	1	23.99	959.86	<0.0001	
Residual	0.1749	7	0.0250			
Lack of fit	0.1602	3	0.0534	14.51	0.0129	
Pure Error	0.0147	4	0.0037			
Cor Total	66.27	16				

Analysis of the influencing factors indicates that the order of their influence on Δ*σ*_so_ from the most significant to the least is: rodless chamber height (*H*)> cylinder inner diameter (*d*_1_)> cylinder wall thickness (*S*).

In summary, based on the response surface methodology (RSM), the quadratic polynomial regression prediction equation for Δ*σ*_so_ established in this study is shown in Equation (15):


$$\begin{array}{c} \begin{array}{c} \Delta \sigma_{\text{so}}=0.0278d_{\text{1}}-0.0194S+0.0187H-0.001d_{\text{1}}S+0.00004d_{\text{1}}H-0.0003SH\\ -0.00001{d_{\text{1}}}^{\text{2}}+0.0037S^{\text{2}}-0.00002H^{\text{2}}-5.01437 \end{array} \end{array}$$ (15)

#### 5.2.2 Static stress difference of cylinder barrel inner wall Δ*σ*_si_.

For the prediction model of the static inner-wall stress peak-to-valley difference (Δ*σ*_si_), the R^2^, adjusted R^2^, and predicted R^2^ values are 0.9977, 0.99488, and 0.9650, respectively, demonstrating strong overall agreement between the fitted quadratic model and the FE-derived responses. The coefficient of variation is 4.18%, indicating relatively limited residual variability in relation to the mean response, while the adequate precision of 47.4294 suggests a sufficiently strong model signal within the investigated parameter range. However, the lack-of-fit test yields an F-value of 31.84 and a p-value of 0.0030, indicating statistically significant discrepancies between the quadratic approximation and the underlying numerical responses. These results suggest that, although the model captures the dominant response trends, its ability to represent localized nonlinear variations remains limited, and its quantitative predictions should be interpreted with appropriate caution.

Significance test and analysis of variance were conducted on the prediction model of the static inner wall stress difference (Δ*σ*_si_). The results are shown in Table 7. The results show that the model’s F-value reaches 341.71 with a P-value less than 0.0001, indicating high statistical significance of the model. The P-values of the cylinder inner diameter (*d*_1_), wall thickness (*S*), rodless chamber height (*H*), interaction term *d*_1_*H*, interaction term *SH*, and quadratic term *H*^2^ are all less than 0.05, demonstrating that each term exerts a significant effect on the response value.

**Table 7:** ANOVA for quadratic model of the Δ*σ*_si._

Source	Sum of Squares	df	Mean Square	F-value	P-value	
Model	300.85	9	33.43	341.71	<0.0001	significant
*d*_1_-*d*_1_	4.12	1	4.12	42.1	0.0003	
*S*-*S*	2.51	1	2.51	25.65	0.0015	
*H*-*H*	187.99	1	187.99	1921.67	<0.0001	
*d*_1_ *S*	0.0030	1	0.0030	0.0309	0.8654	
*d*_1_ *H*	0.9702	1	0.9702	9.92	0.0162	
*SH*	0.5852	1	0.5852	5.98	0.0444	
*d*_1_^2^	0.0188	1	0.0188	0.1918	0.6746	
*S* ^2^	0.0248	1	0.0248	0.2535	0.6300	
*H* ^2^	104.35	1	104.35	1066.70	<0.0001	
Residual	0.6848	7	0.0978			
Lack of fit	0.6573	3	0.2191	31.84	0.0030	
Pure Error	0.0275	4	0.0069			
Cor Total	301.53	16				

Analysis of the influencing factors reveals that the order of their impact on Δ*σ*_si_ from the most significant to the least is: rodless chamber height (*H*)> cylinder inner diameter (*d*_1_)> cylinder wall thickness (*S*).

In summary, the quadratic polynomial regression prediction equation for the static inner wall stress difference (Δ*σ*_si_) of the cylinder barrel is shown in Equation (16):


$$\begin{array}{c} \begin{array}{c} \Delta \sigma_{\text{si}}=-0.1471d_{\text{1}}-1.7841S+0.0393H+0.0012d_{\text{1}}S+0.00008d_{\text{1}}H-0.0006SH\\ +0.0003{d_{\text{1}}}^{\text{2}}+0.0341S^{\text{2}}-0.00003H^{\text{2}}+30.7604 \end{array} \end{array}$$ (16)

#### 5.2.3 Dynamic stress difference of cylinder barrel outer wall Δ*σ*_do_.

For the prediction model of the dynamic outer-wall stress peak-to-valley difference (Δ*σ*_do_), the R^2^, adjusted R^2^, and predicted R^2^ values are 0.9944, 0.9872, and 0.9134, respectively, indicating that the quadratic model captures the principal variation trends of the FE-derived dynamic responses. The coefficient of variation is 7.01%, while the adequate precision reaches 36.0549, exceeding the recommended threshold of 4 and indicating an adequate signal for analyzing parameter effects within the design space. Nevertheless, the lack-of-fit test shows a statistically significant result (F = 35.31, p = 0.0025), suggesting that the quadratic formulation does not fully account for the underlying response characteristics. Therefore, although the model provides a useful approximation of the overall parameter–response relationships, its predictive accuracy may vary across the investigated domain, particularly in regions exhibiting pronounced nonlinear behavior.

Significance test and analysis of variance were performed on the prediction model of the dynamic outer wall stress difference (Δ*σ*_do_). The results are shown in Table 8. The results show that the model’s F-value reaches 138.13 with a P-value less than 0.0001, indicating high statistical significance of the model. The P-values of the cylinder inner diameter (*d*_1_), wall thickness (*S*), rodless chamber height (*H*), and quadratic term *H*^2^ are all less than 0.05, demonstrating that each of these terms has a significant effect on the response value.

**Table 8:** ANOVA for Quadratic model of the Δ*σ*_do._

Source	Sum of Squares	df	Mean Square	F-value	P-value	
Model	6688.41	9	743.16	138.13	<0.0001	significant
*d*_1_-*d*_1_	44.89	1	44.89	8.34	0.0234	
*S*-*S*	40.01	1	40.01	7.44	0.0295	
*H*-*H*	6087.46	1	6087.46	1131.51	<0.0001	
*d*_1_ *S*	9.61	1	9.61	1.79	0.2232	
*d*_1_ *H*	1.92	1	1.92	0.3566	0.5692	
*SH*	24.45	1	24.45	4.55	0.0705	
*d*_1_^2^	20.47	1	20.47	3.81	0.0921	
*S* ^2^	0.6080	1	0.6080	0.1130	0.7466	
*H* ^2^	447.78	1	447.78	83.23	<0.0001	
Residual	37.66	7	5.38			
Lack of fit	36.29	3	12.10	35.31	0.0025	
Pure Error	1.37	4	0.3425			
Cor Total	6726.07	16				

Analysis of the influencing factors indicates that the order of their impact on Δ*σ*_do_ from the most significant to the least is: rodless chamber height (*H*)> cylinder inner diameter (*d*_1_)> cylinder wall thickness (*S*).

In summary, the quadratic polynomial regression prediction equation for the dynamic outer wall stress difference (Δ*σ*_do_) of the cylinder barrel is shown in Equation (17):


$$\begin{array}{c} \begin{array}{c} \Delta \sigma_{\text{do}}=5.7953d_{\text{1}}+8.1185S+0.1972H-0.0689d_{\text{1}}S+0.0001d_{\text{1}}H-0.0041SH\\ -0.0098{d_{\text{1}}}^{\text{2}}+0.1689S^{\text{2}}-0.0001H^{\text{2}}-738.9504 \end{array} \end{array}$$ (17)

#### 5.2.4 Dynamic stress difference of cylinder barrel inner wall Δ*σ*_di_.

For the prediction model of the dynamic inner-wall stress peak-to-valley difference (Δ*σ*_di_), the R^2^, adjusted R^2^, and predicted R^2^ values are 0.9982, 0.9959, and 0.9734, respectively, indicating strong overall fitting performance and favorable internal predictive consistency. The coefficient of variation is 3.75%, suggesting relatively low residual variability, while the adequate precision reaches 54.1991, substantially exceeding the recommended threshold of 4 and confirming an adequate signal for examining the parameter-dependent response trends. However, the lack-of-fit test remains statistically significant (F = 12.96, p = 0.0158), indicating that systematic approximation errors persist despite the high coefficients of determination. Accordingly, the model can be used to characterize the dominant trends in the FE-derived responses, but its quantitative predictions should not be regarded as fully reliable without further verification at independent parameter combinations.

Significance test and analysis of variance were conducted on the prediction model of Δ*σ*_di_ (dynamic inner wall stress difference). The results are shown in Table 9. The results show that the model’s F-value reaches 427.66 with a P-value less than 0.0001, indicating high statistical significance of the model. The P-values of the cylinder inner diameter (*d*_1_), rodless chamber height (*H*), quadratic term *d*_1_^2^, and quadratic term *H*^2^ are all less than 0.05, demonstrating that each of these terms exerts a significant effect on the response value.

**Table 9:** ANOVA for quadratic model of the Δ*σ*_di._

Source	Sum of Squares	df	Mean Square	F-value	P-value	
Model	11847.25	9	1316.36	427.66	<0.0001	significant
*d*_1_-*d*_1_	33.58	1	33.58	10.91	0.0131	
*S*-*S*	1.30	1	1.30	0.4211	0.5371	
*H*-*H*	9475.83	1	9475.83	3078.50	<0.0001	
*d*_1_ *S*	7.10	1	7.10	2.31	0.1726	
*d*_1_ *H*	3.76	1	3.76	1.22	0.3054	
*SH*	0.3660	1	0.3660	0.1189	0.7403	
*d*_1_^2^	21.69	1	21.69	7.05	0.0327	
*S* ^2^	0.0012	1	0.0012	0.0004	0.9847	
*H* ^2^	2265.20	1	2265.20	735.92	<0.0001	
Residual	21.55	7	3.08			
Lack of fit	19.54	3	6.51	12.96	0.0158	
Pure Error	2.01	4	0.5023			
Cor Total	11868.79	16				

Analysis of the influencing factors reveals that the order of their impact on Δ*σ*_di_ from the most significant to the least is: rodless chamber height (*H*)> cylinder inner diameter (*d*_1_)> cylinder wall thickness (*S*).

In summary, the quadratic polynomial regression prediction equation describing the relationship between each influencing factor and the dynamic inner wall stress difference (Δ*σ*_di_) is shown in Equation (18):


$$\begin{array}{c} \begin{array}{c} \Delta \sigma_{di}=5.6663d_{\text{1}}+13.0414S+0.2071H-0.0592d_{\text{1}}S+0.0002d_{\text{1}}H-0.0005SH\\ -0.0101{d_{\text{1}}}^{\text{2}}-0.0076S^{\text{2}}-0.0001H^{\text{2}}-775.6481 \end{array} \end{array}$$ (18)

#### 5.2.5 Model adequacy and statistical evaluation.

The lack-of-fit tests were statistically significant for all four quadratic response surface models. Specifically, the lack-of-fit F-values for Δ*σ*_so_, Δ*σ*_si_, Δ*σ*_do_, and Δ*σ*_di_ were 14.51, 31.84, 35.31, and 12.96, respectively, with corresponding p-values of 0.0129, 0.0030, 0.0025, and 0.0158. These results indicate that the quadratic polynomial formulations do not fully capture the underlying FE response surfaces according to the conventional lack-of-fit criterion.

It should be noted that the response data were generated from finite-element simulations rather than physical experiments. Since repeated deterministic calculations under identical numerical conditions generally exhibit limited variability, the pure-error estimate obtained from replicated design points may be relatively small. Consequently, the lack-of-fit test can be sensitive to systematic discrepancies between the polynomial approximations and the original numerical responses. Nevertheless, this characteristic does not eliminate the observed lack of fit or establish complete model adequacy.

The significant lack of fit may also reflect local nonlinearities or higher-order interactions that are not explicitly represented in the second-order polynomial formulation. In particular, changes in rodless-chamber height may alter the interaction between the boundary-affected regions near the cylinder bottom and piston side, potentially resulting in response variations that cannot be fully reproduced by a single quadratic approximation.

Although the relatively high R² and predicted R² values indicate that the models capture the dominant overall response trends, these metrics do not establish that the models are free from overfitting or systematic approximation errors. Therefore, the quadratic response surfaces are interpreted as approximate FE-based surrogate models within the investigated parameter domain. The models are primarily used to quantify parameter effects, describe interaction trends, and identify configurations associated with pronounced stress non-uniformity. Quantitative predictions at untested configurations, particularly in regions exhibiting substantial local nonlinearities, should be interpreted with caution and verified through additional FE calculations where necessary.

To clarify the basis for ranking the investigated parameters, the relative importance of cylinder inner diameter (*d*_1_), wall thickness (*S*), and rodless-chamber height (*H*) was evaluated using the sums of squares (SS) of their linear main-effect terms in the ANOVA results. Since all three linear terms have one degree of freedom, their rankings based on the sums of squares and corresponding F-values are identical within each response model.

The normalized relative contribution of each linear main effect was calculated as follows:


$$\begin{array}{c} {\text{P}}_{i}=\frac{{\text{SS}}_{i}}{{\text{SS}}_{d_{\text{1}}}{\text{+SS}}_{S}{\text{+SS}}_{H}}\times 100\% \end{array}$$ (19)

where (SS_*d*1_), (SS_*S*_), and (SS_*H*_) represent the sums of squares corresponding to cylinder inner diameter, wall thickness, and rodless-chamber height, respectively.

For the static outer-wall response (Δ*σ*_so_), the relative contributions of cylinder inner diameter, wall thickness, and rodless-chamber height were 2.00%, 1.43%, and 96.58%, respectively. The corresponding values for the static inner-wall response (Δ*σ*_si_) were 2.12%, 1.29%, and 96.59%. Under dynamic loading, the relative contributions were 0.73%, 0.65%, and 98.63% for the outer-wall response (Δ*σ*_do_), and 0.35%, 0.01%, and 99.63% for the inner-wall response (Δ*σ*_di_), respectively.

These results indicate that, within the investigated factor ranges, the relative importance of the linear main effects consistently follows the order of rodless-chamber height, cylinder inner diameter, and wall thickness. In particular, rodless-chamber height makes the largest relative contribution to all four stress peak-to-valley responses. It should be emphasized that these percentages are normalized among the three linear main-effect terms and do not represent their contributions to the total response variance. The interaction and quadratic terms are therefore evaluated separately.

The ANOVA results also indicate that the quadratic term (*H*^2^) is statistically significant for all four responses ((p < 0.0001)), suggesting a pronounced nonlinear dependence on rodless-chamber height. For the static outer-wall response, the (*d*_1_ *H*) interaction is significant ((p = 0.0260)). For the static inner-wall response, both (*d*_1_ *H*) and (*S H*) are significant, with p-values of 0.0162 and 0.0444, respectively. For the dynamic inner-wall response, the quadratic term (*d*_1_^2^) is also significant ((p = 0.0327)), whereas the linear wall-thickness term is not statistically significant ((p = 0.5371)).

Consequently, the reported factor ranking should be interpreted as the relative importance of the linear main effects rather than an invariant local sensitivity ranking over the entire design space. The significance of the quadratic terms further indicates that the effects of the parameters cannot be fully characterized by linear relationships alone.

Moreover, because the quadratic response models exhibit statistically significant lack of fit, the above results should be interpreted as approximate parameter-effect trends within the investigated design space. Their quantitative applicability outside this domain requires additional verification.

### 5.3 Research on the interaction mechanism of influencing factors

#### 5.3.1 Static stress difference of cylinder barrel inner wall and outer wall Δ*σ*_s_.

The static inner and outer wall stress differences (Δ*σ*_si_ and Δ*σ*_so_) respectively reflect the stress states of the inner and outer walls of the cylinder barrel’s rodless chamber under static loads. Fig. 15 and 16 illustrate the interaction relationships of the interaction terms of various factors on Δ*σ*_so_ and Δ*σ*_si_ under static loading conditions, respectively.

Fig 15(a) illustrates the interaction between the cylinder inner diameter (*d*_1_) and wall thickness (*S*) on the static inner wall stress difference (Δ*σ*_si_) when the rodless chamber height (*H*) is fixed at 500 mm.When the cylinder inner diameter is constant, Δ*σ*_si_ decreases with the increase of cylinder wall thickness, showing a negative correlation. The reason is that as the wall thickness increases, the circumferential stress borne by the cylinder decreases significantly, enabling the outer wall to bear the load generated by internal pressure more uniformly. Meanwhile, the increased wall thickness effectively improves the synergistic bearing capacity of circumferential stress and axial stress, thereby reducing Δ*σ*_si_ significantly. When the cylinder wall thickness is constant, Δ*σ*_si_ exhibits a positive correlation with the inner diameter. A larger inner diameter leads to greater circumferential stress under pressure; additionally, the increased inner diameter also causes an increase in bending stress. The combined effect of circumferential stress and bending stress deteriorates the stress state of the cylinder, resulting in an increase in Δ*σ*_si_.

**Fig 15:**
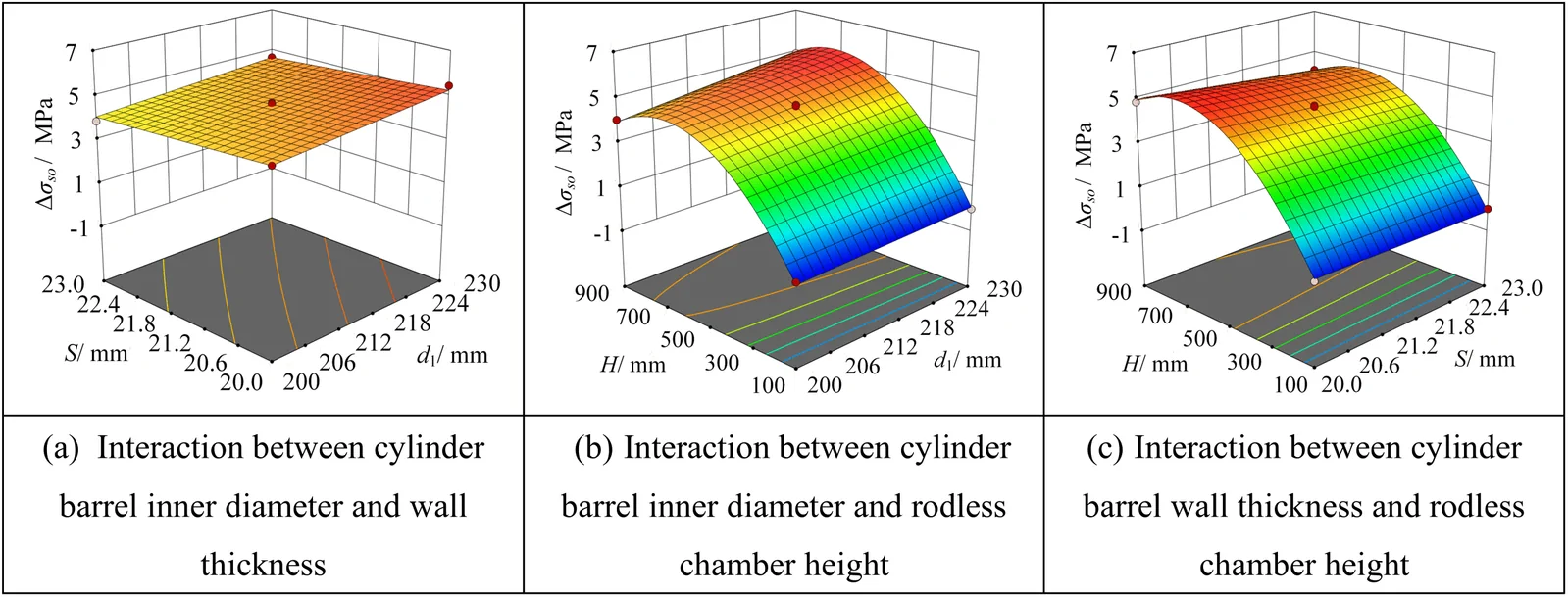
Interaction diagram of interaction terms on Δ*σ*_so_.

As shown in Fig 15(b), when the rodless chamber height (*H*) is constant, the static inner wall stress difference (Δ*σ*_si_) is proportional to the cylinder inner diameter (*d*_1_). The variation degree of Δ*σ*_si_ when the inner diameter is 200 mm is smaller than that when the inner diameter is 230 mm, which corresponds to the phenomenon observed in Fig 15(a). When the inner diameter is constant, within the range of rodless chamber height from 100 mm to 700 mm, Δ*σ*_si_ increases with the increase of rodless chamber height; while in the range from 700 mm to 900 mm, Δ*σ*_si_ decreases as the rodless chamber height increases. The underlying mechanism is as follows: with the increase of rodless chamber height, the internal pressure distribution of the cylinder becomes uneven, and the pressure difference between the bottom and top of the cylinder intensifies—with higher pressure at the bottom and lower pressure at the top—resulting in an increase in Δ*σ*_si_. However, when the rodless chamber height exceeds 700 mm, the cylinder is subjected to the combined action of circumferential stress, radial stress, and axial stress, which leads to an increase in cylinder deflection and a rise in the minimum stress at the middle of the rodless chamber. Consequently, Δ*σ*_si_ decreases accordingly.

Fig 15(c) depicts the interaction between the cylinder wall thickness (*S*) and rodless chamber height (*H*) on the static inner wall stress difference (Δ*σ*_si_) when the cylinder inner diameter (*d*_1_) is fixed at 215 mm. When the rodless chamber height is constant, Δ*σ*_si_ is inversely proportional to the cylinder wall thickness. The variation degree of Δ*σ*_si_ when the wall thickness is 20.0 mm is greater than that when the wall thickness is 23.0 mm, which is consistent with the phenomenon presented in Fig 15(a). When the cylinder wall thickness is constant, the variation law of Δ*σ*_si_ with the rodless chamber height is similar to that in Fig. 15(b), and thus no further elaboration is provided herein.

By comparing Fig 16(a)–(c) with Fig 15(a)–(c), it can be concluded that under static loading conditions, the stress state distributions of the outer and inner walls of the cylinder barrel are basically consistent. Since the static loading method simulates the normal supporting working state of the oil cylinder under mine conditions or the state of terrain creep, the load borne by the cylinder barrel is relatively stable, resulting in relatively uniform stress distribution without local excessive stress concentration.

**Fig 16:**
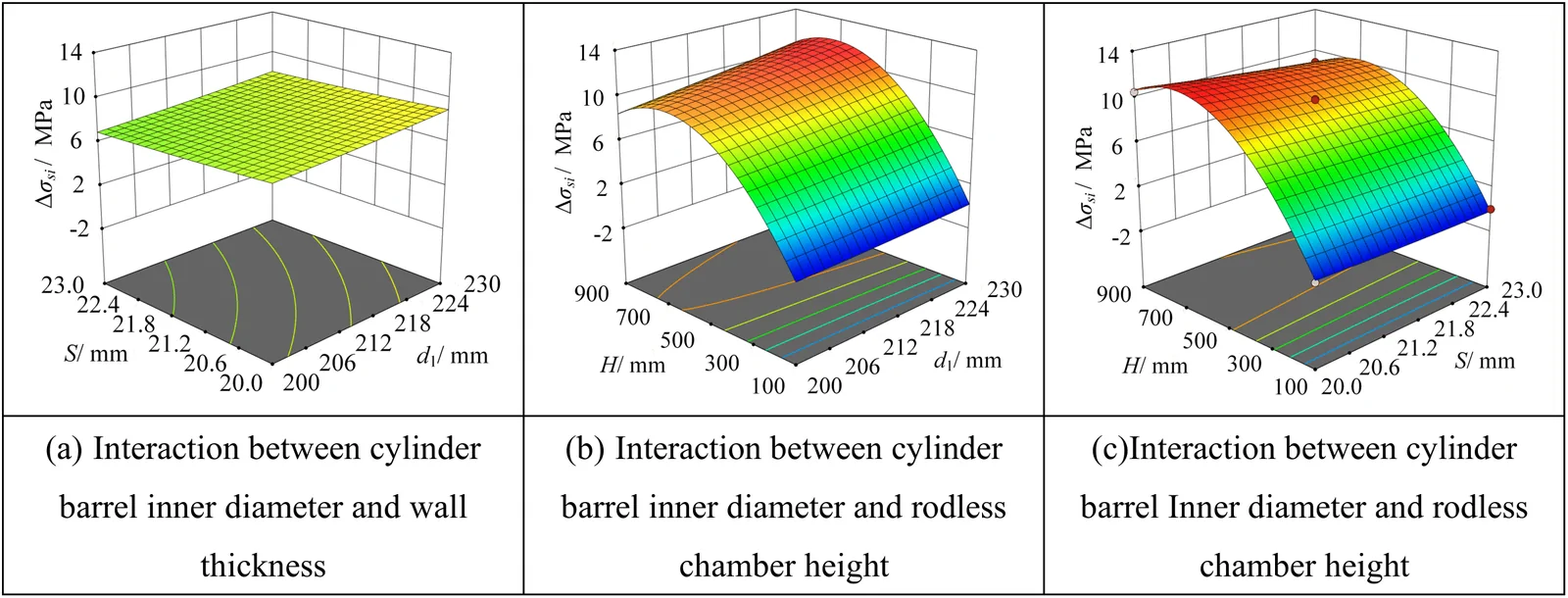
Interaction diagram of interaction terms on Δ*σ*_si_.

#### 5.3.2 Dynamic stress difference of cylinder barrel inner wall and outer wall Δ*σ*_d_.

The dynamic inner and outer wall stress differences (Δ*σ*_di_ and Δ*σ*_do_) respectively reflect the stress states of the inner and outer walls of the cylinder barrel’s rodless chamber under dynamic loads. Fig 17 and Fig 18 illustrate the interaction relationships of the interaction terms of various factors on Δ*σ*_do_ and Δ*σ*_di_ under dynamic loading conditions, respectively.

**Fig 17:**
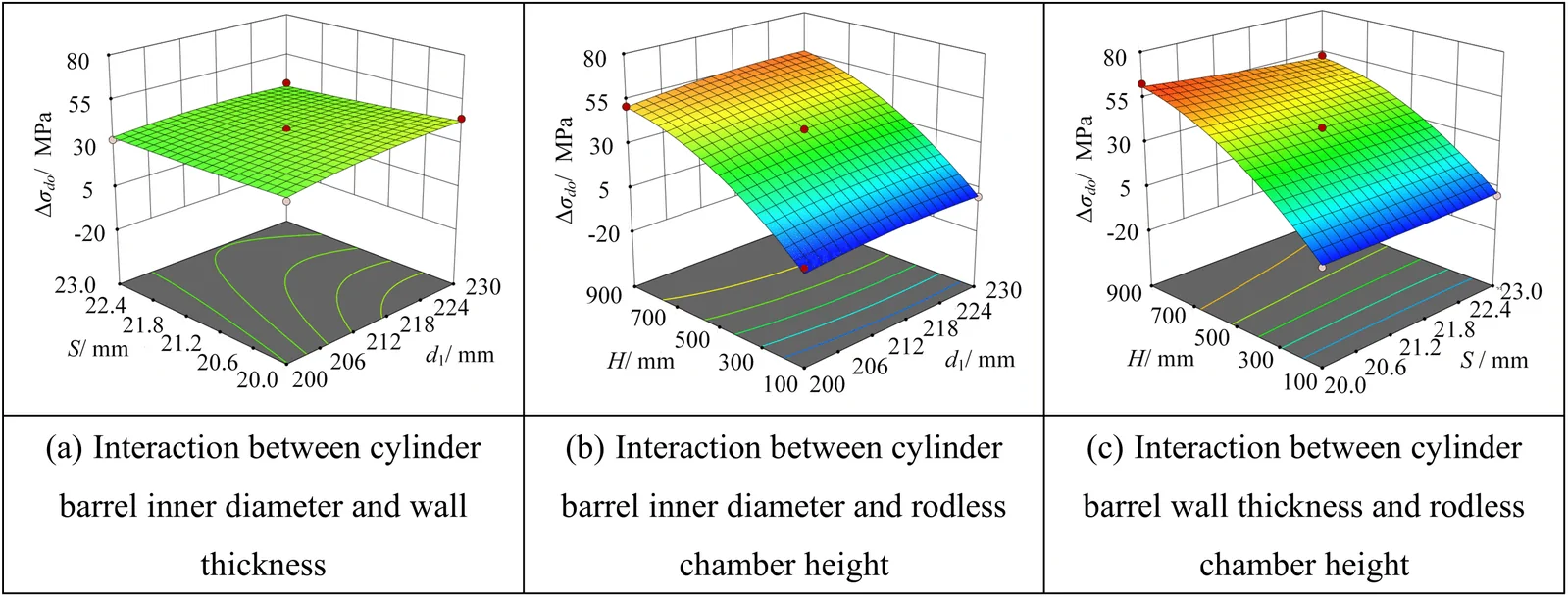
Interaction diagram of interaction terms on Δ*σ*_do._

**Fig 18:**
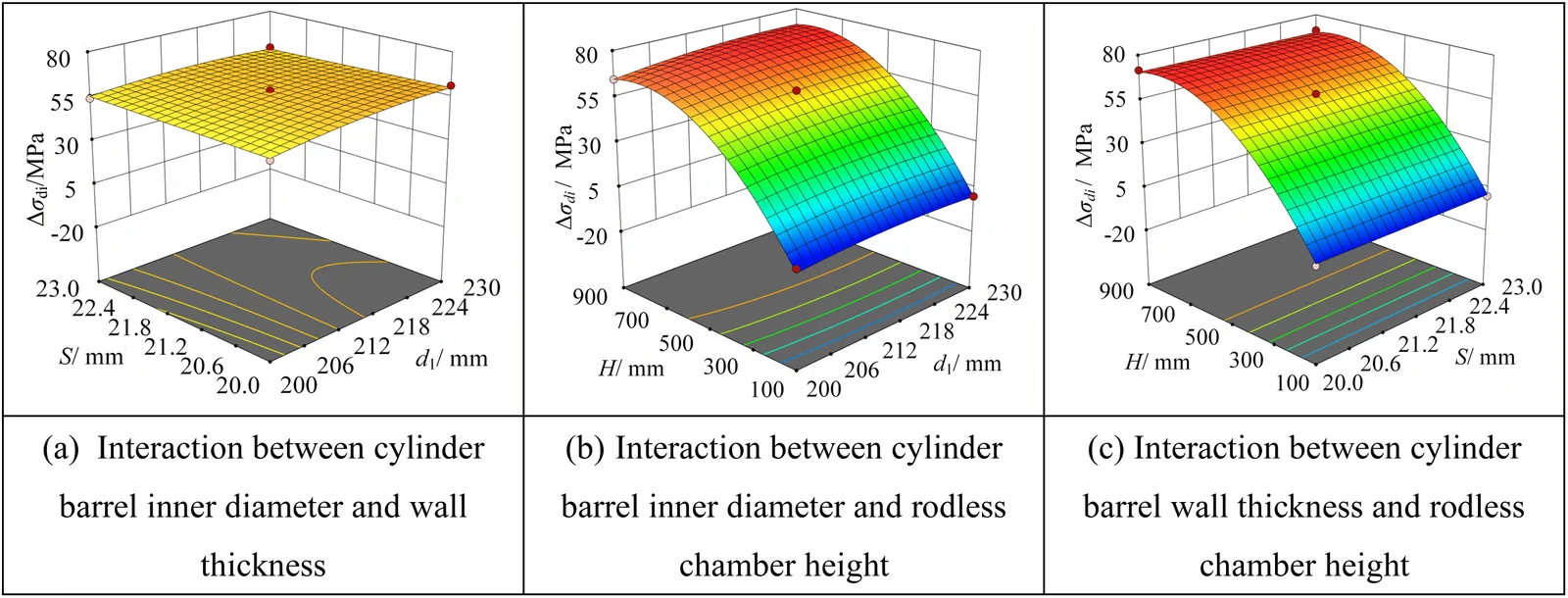
Interaction diagram of interaction terms on Δ*σ*_di_.

As shown in Fig 17(a), when the cylinder inner diameter (*d*_1_) is constant, the dynamic outer wall stress difference (Δ*σ*_do_) is negatively correlated with the cylinder wall thickness (*S*). The variation degree of Δ*σ*_do_ when the wall thickness is 20.0 mm is greater than that when the wall thickness is 23.0 mm. When the cylinder wall thickness is constant, within the range of the cylinder inner diameter from 200 mm to 218 mm, Δ*σ*_do_ increases with the increase of the inner diameter; while in the range from 218 mm to 230 mm, Δ*σ*_do_ is negatively correlated with the inner diameter. The reason is that as the inner diameter increases, the circumferential stress and bending stress generated under pressure both increase. The combined effect of these two stresses deteriorates the stress state of the cylinder, leading to an increase in Δ*σ*_do_. However, when the inner diameter exceeds 218 mm, the difference from static mechanics lies in that the dynamic load borne is an impact load. Under the action of the impact load, the cylinder deflection changes significantly, resulting in an increase in the stress borne by the middle of the rodless chamber and thus a decrease in Δ*σ*_do_.

Fig 17(b) depicts the interaction between the cylinder inner diameter (*d*_1_) and rodless chamber height (*H*) on the dynamic outer wall stress difference (Δ*σ*_do_) when the cylinder wall thickness (*S*) is fixed at 21.5 mm. When the rodless chamber height is constant, within the range of the cylinder inner diameter from 200 mm to 218 mm, Δ*σ*_do_ increases with the increase of the inner diameter; while in the range from 218 mm to 230 mm, Δ*σ*_do_ is negatively correlated with the inner diameter, which corresponds to the phenomenon observed in Fig. 17(a). When the cylinder inner diameter is constant, as the rodless chamber height increases, the axial stress of the cylinder increases under the action of the impact load. This leads to an increase in the maximum stress of the cylinder, consequently resulting in a corresponding increase in Δ*σ*_do_.

As shown in Fig 17(c), when the rodless chamber height (*H*) is constant, the dynamic outer wall stress difference (Δ*σ*_do_) is inversely proportional to the cylinder wall thickness (*S*). The variation degree of Δ*σ*_do_ when the wall thickness is 20.0 mm is greater than that when the wall thickness is 23.0 mm, which is consistent with the phenomenon presented in Fig 17(a). When the cylinder wall thickness is constant, the variation law of Δ*σ*_do_ with the rodless chamber height is similar to that in Fig 17(b), and thus no further elaboration is provided herein.

By comparing Fig 18(a)–(c) with Fig 18(a)–(c), it can be found that the variation rules of the dynamic inner wall stress difference (Δ*σ*_di_) affected by the cylinder inner diameter (*d*_1_) and wall thickness (*S*) are basically consistent with those of the outer wall (Δ*σ*_do_). However, under dynamic loading conditions, there is a difference in the influence of the rodless chamber height (*H*) on Δ*σ*_di_ compared with Δ*σ*_do_: within the range of rodless chamber height from 100 mm to 750 mm, Δ*σ*_di_ increases with the increase of the rodless chamber height; while in the range from 750 mm to 900 mm, Δ*σ*_di_ is negatively correlated with the rodless chamber height. The reason for the difference in the variation rules affected by the rodless chamber height lies in that when the stress wave propagates from the inner wall to the outer wall of the cylinder, the propagation of the stress wave is delayed. This delay results in a more uniform stress distribution on the outer wall than on the inner wall, leading to the distinct variation trends between Δ*σ*_di_ and Δ*σ*_do_.

### 5.4 Identification of key structural parameters affecting M-shaped stress characteristics

To explore the structural parameters corresponding to the most obvious “M”-shaped characteristic of the cylinder stress state distribution, the maximum values of the four response variables were solved using the response prediction models. The obtained optimal structural parameters are as follows: cylinder inner diameter (*d*_1_) = 230 mm, cylinder wall thickness (*S*) = 20 mm, and rodless chamber height (*H*) = 795 mm. As indicated by the prediction equations, under these structural parameters, the static outer wall stress difference (Δ*σ*_so_) is 5.896 MPa, the static inner wall stress difference (Δ*σ*_si_) is 12.658 MPa, the dynamic outer wall stress difference (Δ*σ*_do_) is 60.301 MPa, and the dynamic inner wall stress difference (Δ*σ*_di_) is 73.984 MPa.

After modeling with the aforementioned structural parameters, finite element analysis (FEA) was performed by applying static and dynamic loads respectively. The stress state distributions of the inner and outer walls of the cylinder are shown in Fig 19. As can be seen from Fig 19, under these structural parameters, the finite element analysis results are as follows: static outer wall stress difference (Δ*σ*_so_) = 5.31 MPa, static inner wall stress difference (Δ*σ*_si_) = 11.42 MPa, dynamic outer wall stress difference (Δ*σ*_do_) = 56.57 MPa, and dynamic inner wall stress difference (Δ*σ*_di_) = 72.13 MPa. A comparison between the FEA results and the predicted values shows that the maximum error is 9.93% and the minimum error is 2.51%, with all errors within the range of 10%. It should be emphasized that this comparison represents numerical verification of the RSM-predicted responses rather than direct experimental validation of the critical configuration. The full-scale experimental validation reported in the previous section was performed using the reference hydraulic-column configuration, which differs from the configuration identified by the RSM analysis. Therefore, the present results support the use of the RSM models for screening potentially unfavorable parameter combinations, while the quantitative predictions at untested configurations should be interpreted with appropriate caution.

**Fig 19:**
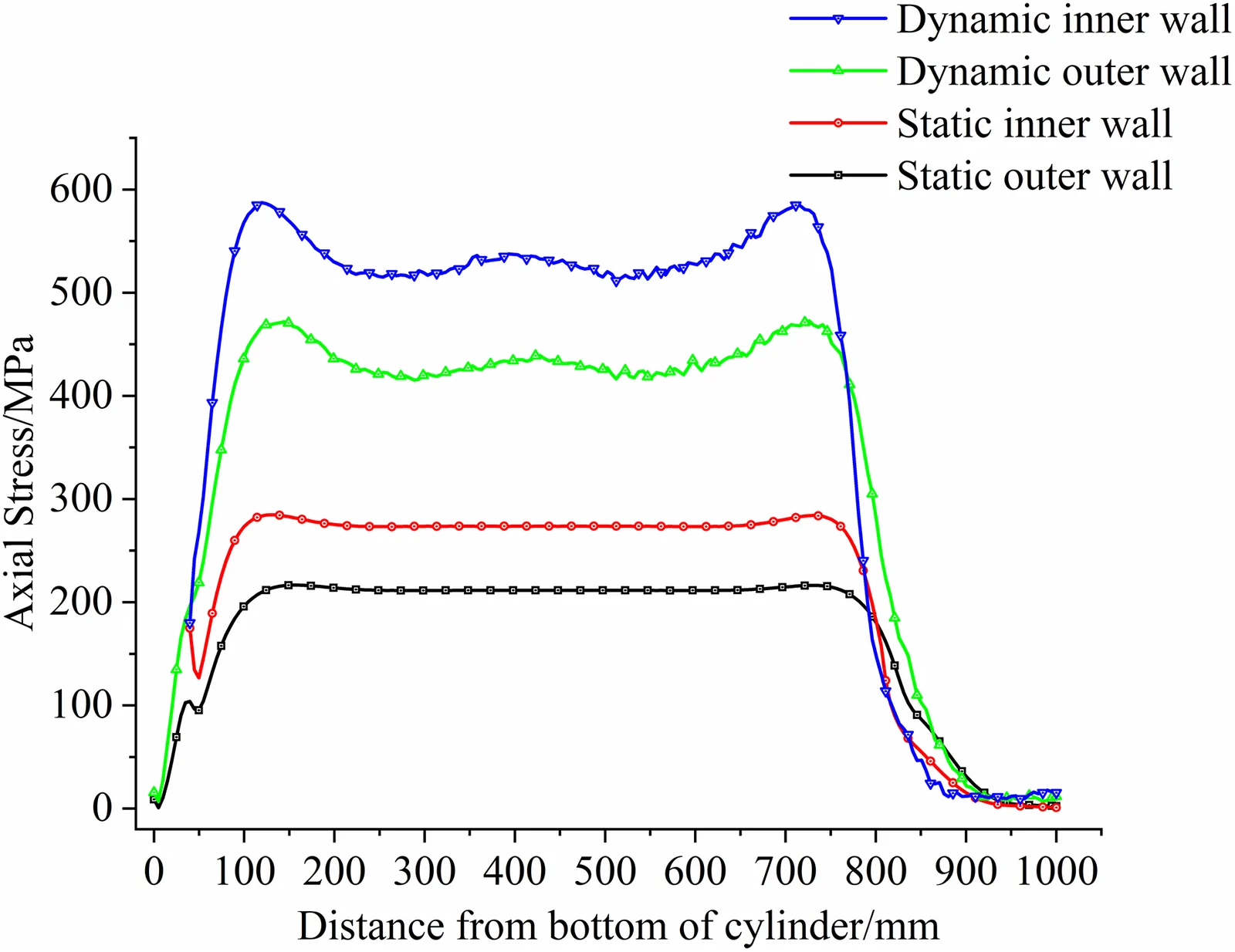
Finite element analysis results of predicted data.

The response surface analysis results show that the parameter combination of *d*_1_ = 230 mm, *S* = 20 mm, and *H* = 795 mm corresponds to a significant axial M-shaped stress distribution of the cylinder barrel. It should be clarified that this parameter combination does not represent the optimal stress state or the structurally safest design scheme. Instead, it denotes a critical structural configuration that substantially aggravates axial stress inhomogeneity, which can be used to identify unfavorable parameter matches and dangerous working conditions that should be deliberately avoided in the structural design and strength verification of hydraulic columns.

The established response surface model quantifies the mapping relationship between key geometric parameters and M-shaped stress characteristics, and clarifies the multi-parameter coupling mechanism affecting the axial stress uniformity of hydraulic columns. This model can predict and avoid adverse parameter combinations that induce severe stress concentration and non-uniform stress distribution within a certain range. It provides a reliable theoretical basis and design reference for optimizing the structural configuration, improving axial stress distribution, and enhancing the service stability and structural reliability of hydraulic pillar components.

### 5.5 Formation mechanism of the M-shaped stress distribution

The M-shaped axial stress distribution along the hydraulic-column cylinder barrel can be explained by the combined effects of internal hydraulic pressure and localized boundary constraints. Thick-walled cylinder theory describes the fundamental pressure-induced stress state, while rotational-shell theory accounts for the additional axial stress disturbances caused by the constraints near the cylinder bottom and piston side. These boundary effects gradually decay toward the intermediate region, resulting in relatively high axial stresses near both ends and a lower axial stress in the middle, thereby providing a mechanical explanation for the M-shaped distribution.

The finite-element (FE) results exhibit similar axial stress characteristics, with two relatively high-stress regions near the constrained boundaries and a lower-stress intermediate region. Experimental measurements along the outer surface of the cylinder barrel provide supporting evidence for the spatial response trends predicted by the FE model. Their agreement supports the numerical model under the tested conditions, although the inner-wall axial stress distribution remains a numerical prediction rather than a directly measured result.

Under transient loading, the axial stress response may be further influenced by structural inertia, time-dependent load transfer, and boundary constraints. These effects may contribute to the differences between static and dynamic axial stress distributions. Nevertheless, the available results do not directly establish specific stress-wave propagation or reflection mechanisms, which require further time-resolved investigation.

The response surface methodology (RSM) analysis quantifies the effects of cylinder inner diameter, wall thickness, and rodless-chamber height on the FE-derived axial stress peak-to-valley differences. In particular, variations in rodless-chamber height change the separation between the two boundary-affected regions, thereby influencing the M-shaped axial stress distribution. Since the RSM models are based on FE-generated data and exhibit statistically significant lack of fit, their predictions should be interpreted as approximate trends within the investigated parameter range rather than independent mechanistic validation.

At small rodless-chamber heights, the axial stress distribution may transition from a double-peaked M-shaped profile to a single-peaked pattern as the boundary-induced axial stress disturbances increasingly interact. The disappearance of the characteristic two-peak–one-valley structure indicates that the M-shaped feature is no longer identifiable under the adopted criterion, rather than an absence of axial stress variation or local stress concentration. Therefore, the disappearance of the M-shaped feature should be distinguished from an actual reduction in axial stress non-uniformity. Furthermore, such a transition does not necessarily imply improved structural safety, which must be assessed using the absolute stress levels and appropriate material strength criteria.

From an engineering perspective, the significance of the M-shaped axial stress distribution lies not only in the axial stress magnitude but also in its spatial non-uniformity. The cylinder-barrel material, 35CrMnSiA steel, has a specified yield strength of 1275 MPa. Under dynamic loading, the maximum reported outer-wall and inner-wall axial stresses were 375.03 and 480.45 MPa, respectively, with the latter corresponding to approximately 37.7% of the specified yield strength. Although these axial stress values are lower than the yield strength, the occurrence of yielding must be assessed using the corresponding von Mises equivalent stress. Therefore, the present study focuses primarily on axial stress non-uniformity rather than directly predicting material yielding or structural failure.

To quantify this non-uniformity, the axial stress peak-to-valley difference was adopted as the principal response variable where a distinguishable M-shaped profile exists. A larger difference indicates more pronounced axial stress variations, which may be accompanied by non-uniform strain and deformation due to the different boundary constraints. Such deformation differences may influence local radial expansion, deformation compatibility, and sealing reliability under repeated loading. However, the axial stress peak-to-valley difference cannot directly quantify radial displacement or permanent deformation. Accordingly, the RSM-identified configuration (230 mm inner diameter, 20 mm wall thickness, and 795 mm rodless-chamber height) represents a potentially unfavorable axial stress distribution condition rather than a confirmed yielding or structural failure limit.

## 6. Conclusions

Aiming at the non-uniform axial stress distribution of the hydraulic column under static and impact loads, this study investigates the stress formation mechanism of the cylinder and the influences of major geometric and operational parameters by combining the mechanism model, finite element analysis, loading tests, and response surface methodology (RSM). The main conclusions are summarized as follows:

(1) A stress analysis model for the hydraulic column is established based on the thick-walled cylinder theory and the edge effect theory of rotating shells. Theoretical analysis, finite element simulation, and experimental results consistently reveal that the cylinder presents an M-shaped axial stress distribution under both static and dynamic loads, with higher stress at both ends and lower stress in the middle region. This distribution is jointly dominated by the fundamental stress induced by internal hydraulic pressure and the local additional edge stress generated by boundary constraints at the cylinder bottom and piston side. The additional edge stress gradually decays with the increase of axial distance from the boundaries, forming a typical peak-valley-peak spatial stress redistribution characteristic.
(2) The peak-valley stress differences of the inner and outer walls under static and dynamic working conditions are adopted to quantify the axial stress inhomogeneity of the cylinder barrel. The response relationships between the key structural parameters (inner diameter, wall thickness, and rodless chamber height) and the peak-valley stress difference are established. Within the parameter range of this study, the influencing significance of the three factors is ranked as follows: rodless chamber height presents the most prominent effect, followed by cylinder inner diameter, while wall thickness has a relatively weak influence. The results indicate that the local stress inhomogeneity of the cylinder is comprehensively controlled by structural dimensions and working positions.
(3) Within the investigated parameter range, the combination of a cylinder inner diameter of 230 mm, wall thickness of 20 mm, and rodless chamber height of 795 mm corresponds to a significant M-shaped stress inhomogeneity. This configuration should be regarded as a critical unfavorable structure requiring priority verification rather than an optimal engineering design. In practical engineering design, for hydraulic columns with large cylinder diameter and thin wall, priority should be given to evaluating the local stress, structural stiffness, and fatigue safety near the cylinder bottom, piston side, and external welding joints. The rodless chamber height of 795 mm corresponds to a critical working state, which should be emphasized in static and dynamic safety verification throughout the full stroke. The proposed method can screen unfavorable structural parameter combinations and critical working conditions, providing a theoretical reference for the structural design of impact-resistant hydraulic columns.

The analytical and numerical models in this study adopt assumptions of material homogeneity, isotropy, and linear elasticity, while simplifying the hydraulic medium characteristics, contact behaviors, and dynamic impact processes. Furthermore, the critical configurations identified via the response surface method have not yet been verified through targeted full-scale experiments. Therefore, the relevant conclusions are only applicable within the ranges of material properties, load conditions, boundary constraints, and structural parameters adopted in this work. Future studies will further consider material nonlinearity, hydraulic medium compressibility, fluid–structure interaction effects, as well as repetitive or random impact loads and time-varying contact behaviors. Full-scale static and dynamic tests targeting the critical configurations will also be performed to validate the engineering applicability of the proposed model.

## Supporting information

S1 Table Impact dynamic loading test. (XLSX)

S2 Table Satic loading tests. (XLSX)

S1 File Numerical modeling. (RAR)
